# Supplementary material for: An mRNA-display derived cyclic peptide scaffold reveals the substrate binding interactions of an N-terminal cysteine oxidase
Source: Nat Commun. 2025 May 22;16:4761. doi: 10.1038/s41467-025-59960-3 (PMC12098869; doi:10.1038/s41467-025-59960-3)

Supporting information

An mRNA-display derived cyclic peptide scaffold reveals the substrate binding interactions of an N-terminal cysteine oxidase

Yannasittha Jiramongkol[1,2], Karishma Patel[1,3], Jason Johansen-Leete[1], Joshua W.C. Maxwell[1,4], Pat Chang[5], Jonathan J. Du[5], Toby Passioura[6], Kristina M. Cook[7], Richard J. Payne[1,4], Mark D. White[1*]

[1]School of Chemistry, The University of Sydney, NSW 2006 Australia
[2]Faculty of Science, Charles Perkins Centre, The University of Sydney, NSW 2006, Australia
[3]School of Life and Environmental Sciences, The University of Sydney, NSW 2006 Australia
[4]Australian Research Council Centre of Excellence for Innovations in Peptide and Protein Science, The University of Sydney, NSW 2006, Australia
[5] School of Pharmacy, The University of Sydney, NSW 2006 Australia
[6] Sydney Analytical Core Research Facility, The University of Sydney, NSW 2006, Australia
[7]Faculty of Medicine and Health, Charles Perkins Centre, The University of Sydney, NSW 2006, Australia

*Corresponding Author: Dr Mark D. White. Address: School of Chemistry, University of Sydney, NSW 2006, Australia. Email: mark.white@sydney.edu.au

Supplementary figures

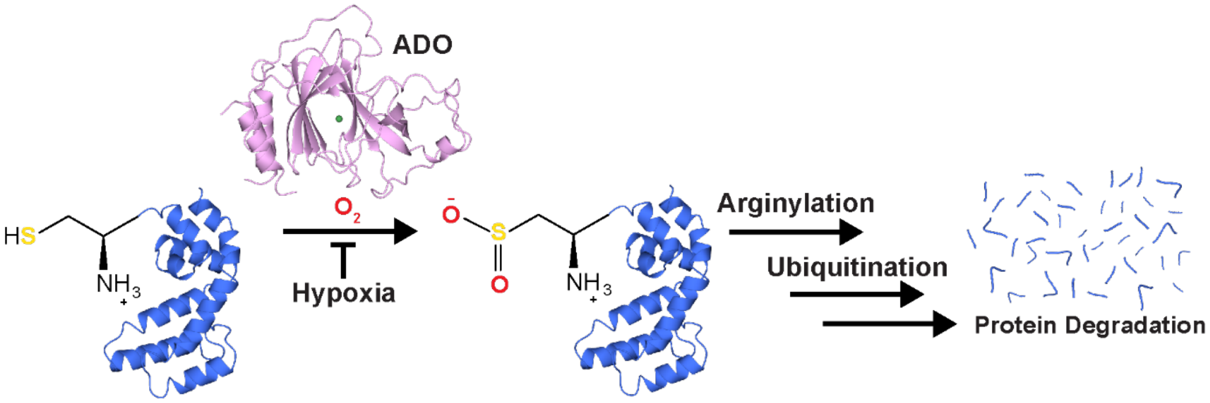

Supplementary Figure 1. A scheme highlighting the role of NCOs (such as ADO) in the Cys-branch of the N-degron pathway. NCOs (such as ADO) catalyse the O_2-dependent sulfinylation of co- or post-translationally exposed Nt-Cys residues, which promotes degradation of the target protein following subsequent arginylation and ubiquitination.

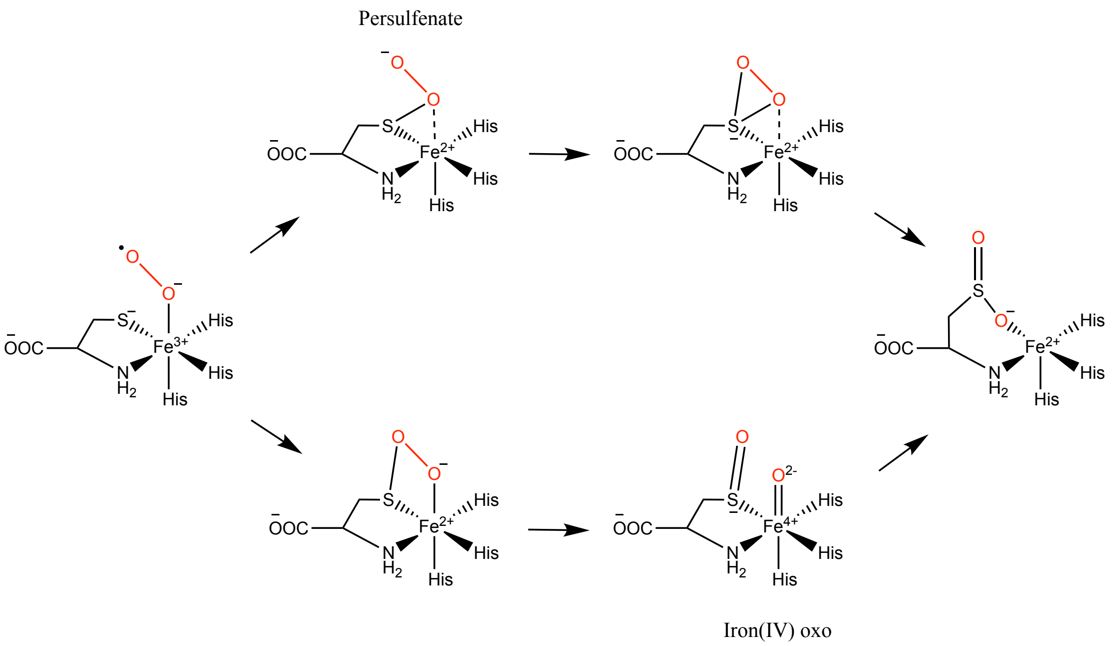

Supplementary Figure 2. Two reaction pathways proposed for CDO. Initial reaction with the proximal oxygen atom results in formation of persulfenate intermediate through a concerted mechanism (top route). Initial reaction with the distal oxygen atom results in formation of an iron(IV) oxo species through a step wise mechanism (bottom route).

ADO Construct	Amino acid sequence
pQE80L-Navi-ADO	**MSGLNDIFEAQKIEWHEGHHHHHHGS**MPRDNMASLIQRIARQACLTFRGSGGGRGASD<br>RDAASGPEAPMQPGFPENLSKLKSLLTQLRAEDLNIAPRKATLQPLPPNLPPVTYMHIYETD<br>GFSLGVFLLKSGTSIPLHDHPGMHGMLKVLYGTVRISCMDKLDAGGGQRPRALPPEQQFEP<br>PLQPREREAVRPGVLRSRAEYTEASGPCILTPHRDNLHQIDAVEGPAAFLDILAPPYDPDDG<br>RDCHYYRVLEPVRPKEASSSACDLPREVWLLETPQADDFWCEGEPYPGPKVFP
pET28a-ADO	**MGSSHHHHHHSSGLVPRGSH**MPRDNMASLIQRIARQACLTFRGSGGGRGASDRDAASGP<br>EAPMQPGFPENLSKLKSLLTQLRAEDLNIAPRKATLQPLPPNLPPVTYMHIYETDGFSLGVF<br>LLKSGTSIPLHDHPGMHGMLKVLYGTVRISCMDKLDAGGGQRPRALPPEQQFEPPLQPRER<br>EAVRPGVLRSRAEYTEASGPCILTPHRDNLHQIDAVEGPAAFLDILAPPYDPDDGRDCHYYR<br>VLEPVRPKEASSSACDLPREVWLLETPQADDFWCEGEPYPGPKVFP
pETDuet-TEV-ADO	**MGSSHHHHHHSQDPENLYFQS**MPRDNMASLIQRIARQACLTFRGSGGGRGASDRDAASG<br>PEAPMQPGFPENLSKLKSLLTQLRAEDLNIAPRKATLQPLPPNLPPVTYMHIYETDGFSLGV<br>FLLKSGTSIPLHDHPGMHGMLKVLYGTVRISCMDKLDAGGGQRPRALPPEQQFEPPLQPRE<br>REAVRPGVLRSRAEYTEASGPCILTPHRDNLHQIDAVEGPAAFLDILAPPYDPDDGRDCHYY<br>RVLEPVRPKEASSSACDLPREVWLLETPQADDFWCEGEPYPGPKVFP

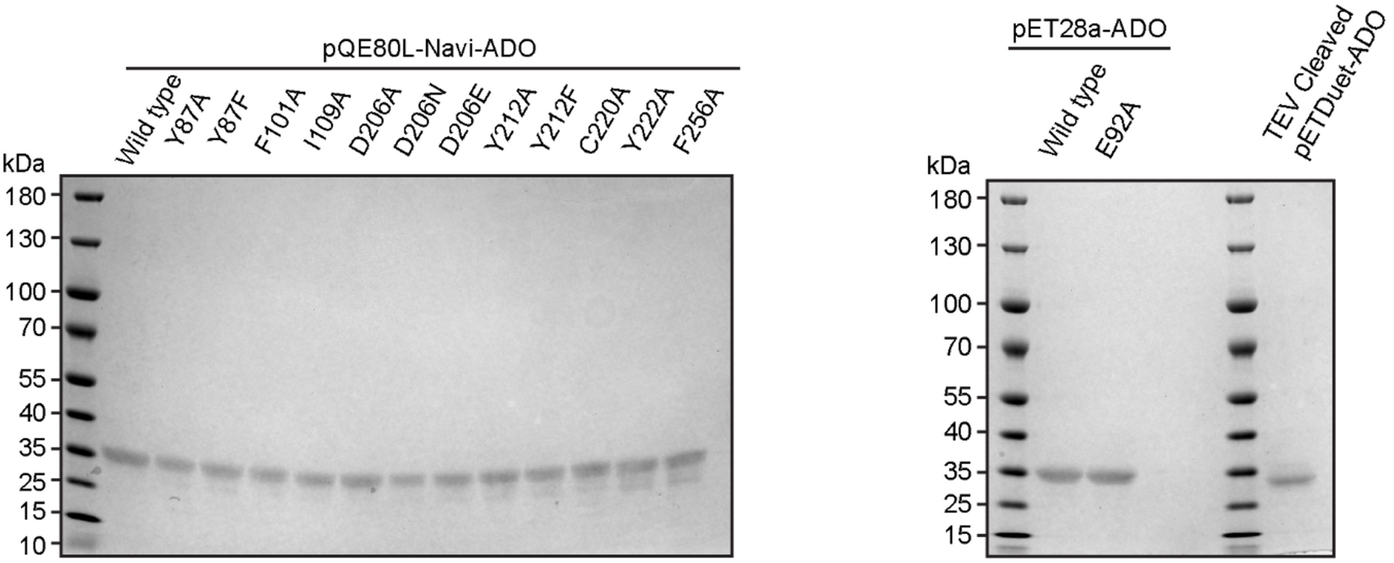

Supplementary Figure 3. ADO protein purification. Three ADO constructs were used during this investigation, all of which possessed an N-terminal poly-histidine tag with different features. pQE80L-Navi-ADO contained an Avi tag, which facilitated site specific biotinylation for RaPID selection and binding analysis, pET28a-ADO contained a thrombin cleavage site, which was retained as a linker during activity assays, and pETDuet-TEV-ADO contained a TEV cleavage site, which used to facilitate tag removal during crystallisation studies. Top: the amino acid compositions of each (wild type) ADO construct. Tags are highlighted in bold. Bottom: SDS PAGE gels depicting the size and purity of each construct and variant. Various mutants were generated, which exhibited the same size and purity as wild type ADO.

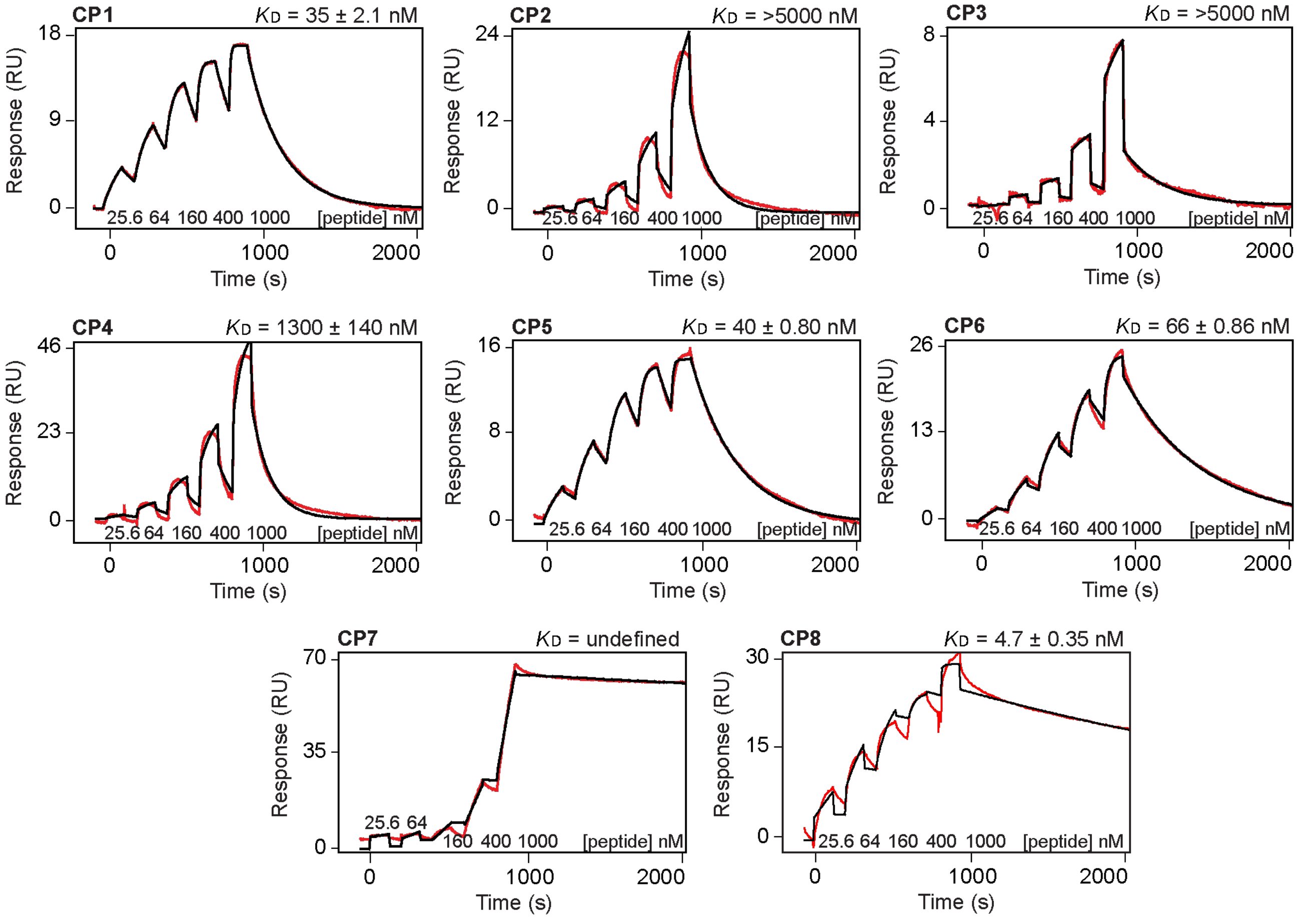

Supplementary Figure 4. Biophysical analysis of the CPs identified through RaPID. Representative SCK SPR sensorgrams of the interactions of the CPs with ADO. The sensorgram is shown in red and the fit to the data is shown in black. The concentrations of CPs used in the titrations and the K_D value is shown (K_D given as the geometric mean of a minimum of three independent SPR measurements (n=3) with standard error). Source data are provided as Source Data file.

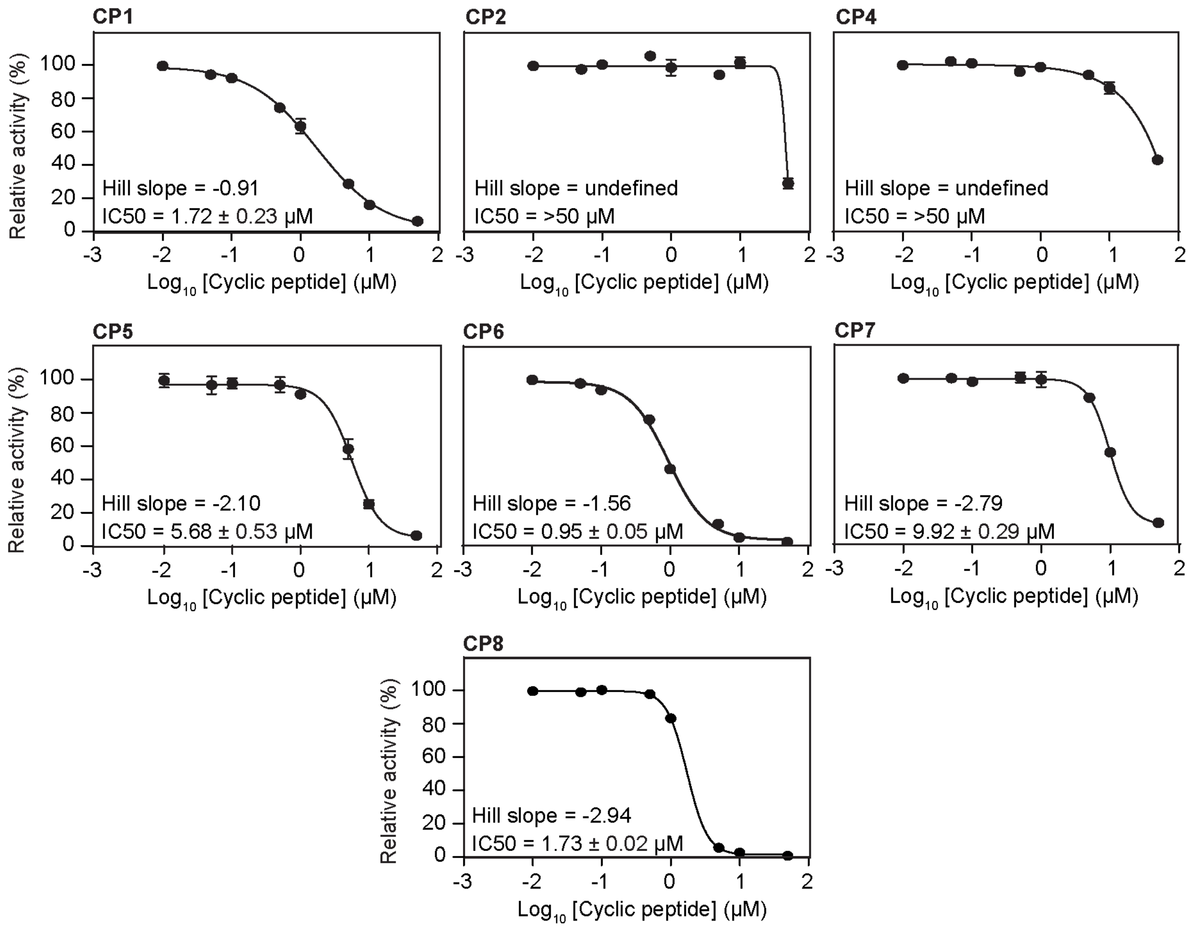

Supplementary Figure 5. Inhibitor profiling of the CPs identified through RaPID. Dose-response curve with IC_{50} values for each CP. The average of three independent experiments (n=3) is shown (error bars show the standard error). Source data are provided as Source Data file.

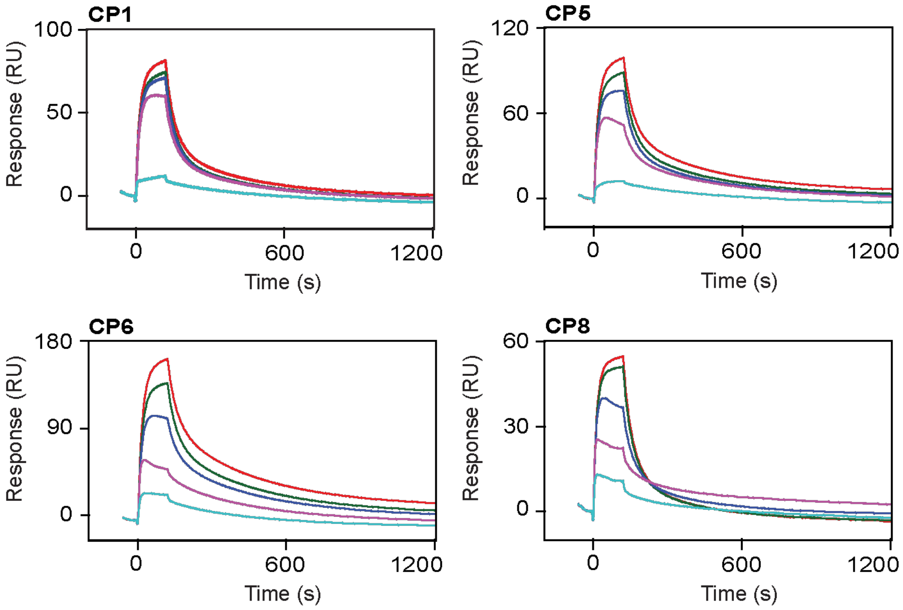

Supplementary Figure 6. SPR competitive binding assays. Native $RGS5^{FL}$ protein alone (5 µM; red) or in the presence of different concentrations of CPs (0.005 µM; green, 0.05 µM; blue, 0.5 µM; purple) were injected over immobilised ADO in separate cycles. CPs alone (0.5 µM; teal) were injected in the absence of $RGS5^{FL}$ protein as a minimal threshold response. CP concentration inversely correlates with $RGS5^{FL}$ association.

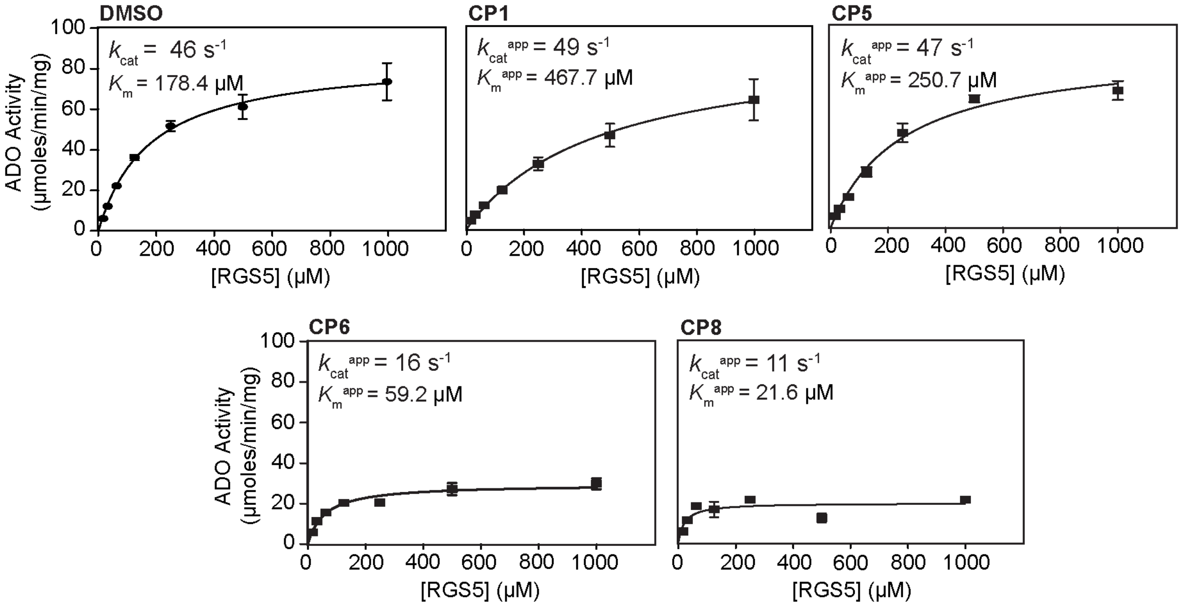

Supplementary Figure 7. Michaelis-Menten kinetic plots of ADO activity in the absence and presence of CP1, 5, 6 and 8. CPs were added at their IC_{50} concentration. Activity was measured under aerobic conditions at 37 °C. The average of three independent experiments (n=3) is shown (error bars show the standard error). Source data are provided as Source Data file.

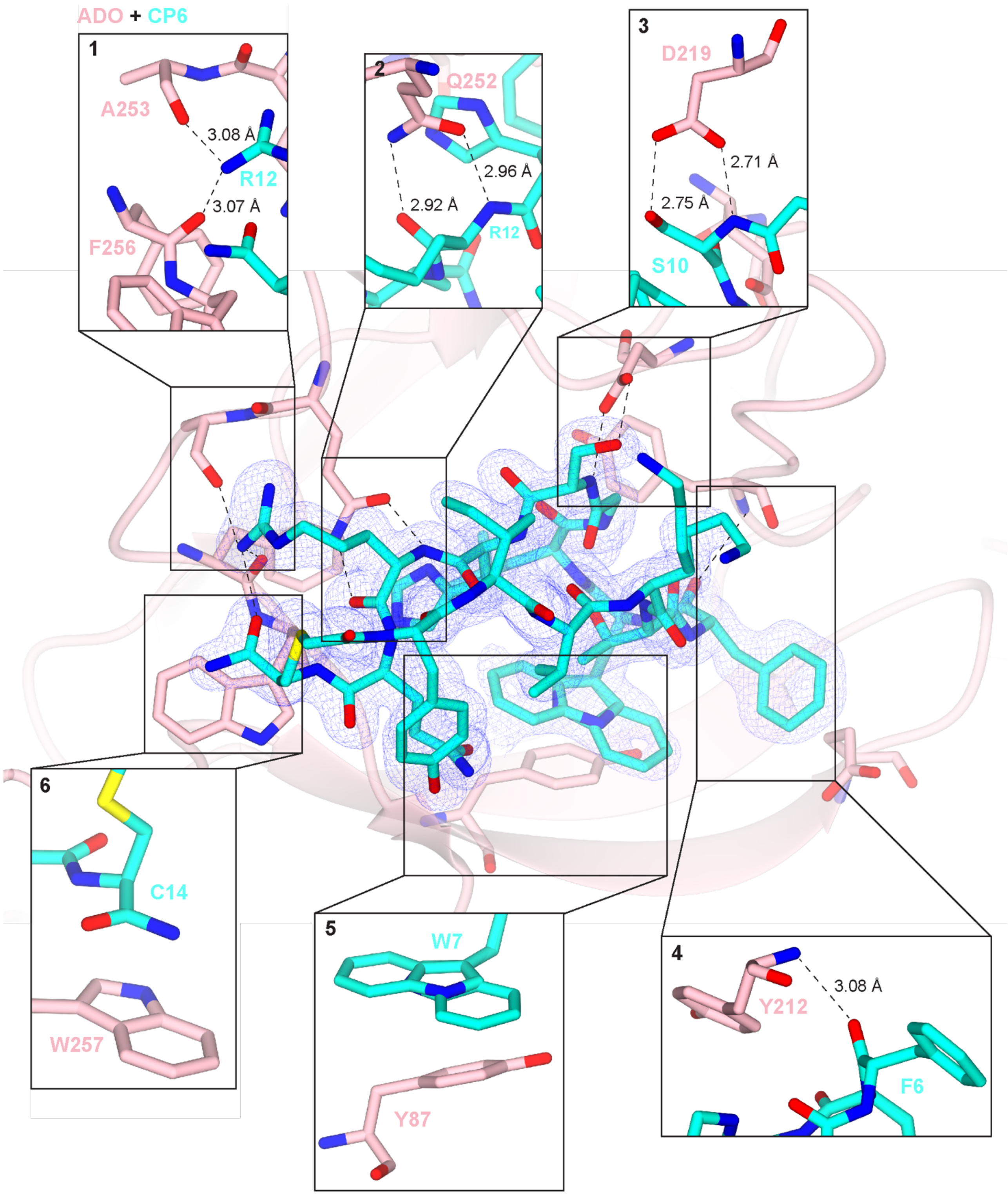

Supplementary Figure 8. The intermolecular interactions between ADO and CP6. CP6 (cyan) interacts with ADO (pink) through multiple hydrogen bonds (boxes 1-4; black dashes) and pi-stackings (boxes 5 and 6). Red denotes hydroxyl group. Blue denotes amine group. 2Fo-Fc electron density is shown as a blue mesh contoured at 1 sigma. The images were generated using CCP4MG.

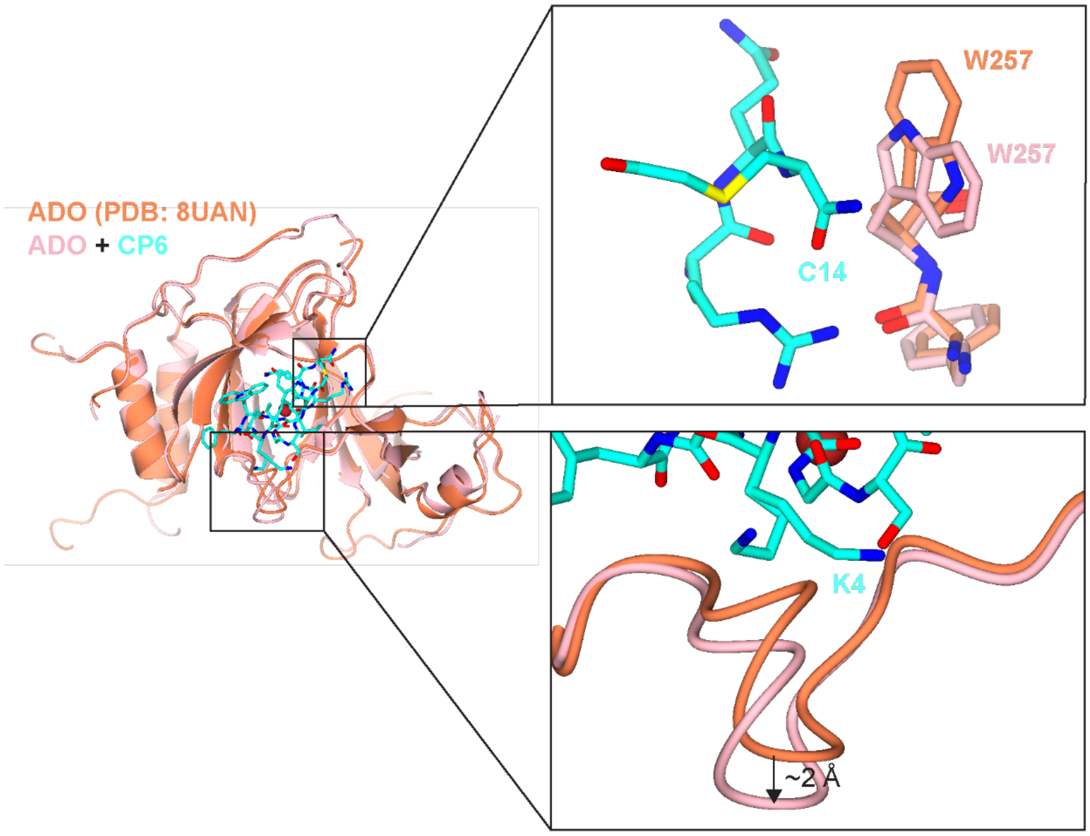

Supplementary Figure 9. The superimposed structures of CP6-free (orange; PDB:8UAN) and CP6-bound (pink; PDB:9DXU) cobalt-incorporated ADO. CP6 (cyan) causes the side chain of ADO-W257 to rotate and stack with the amide of CP6-C14 (top boxed insert) and ADO loop 212-220 to move ~2 Å (bottom boxed insert).

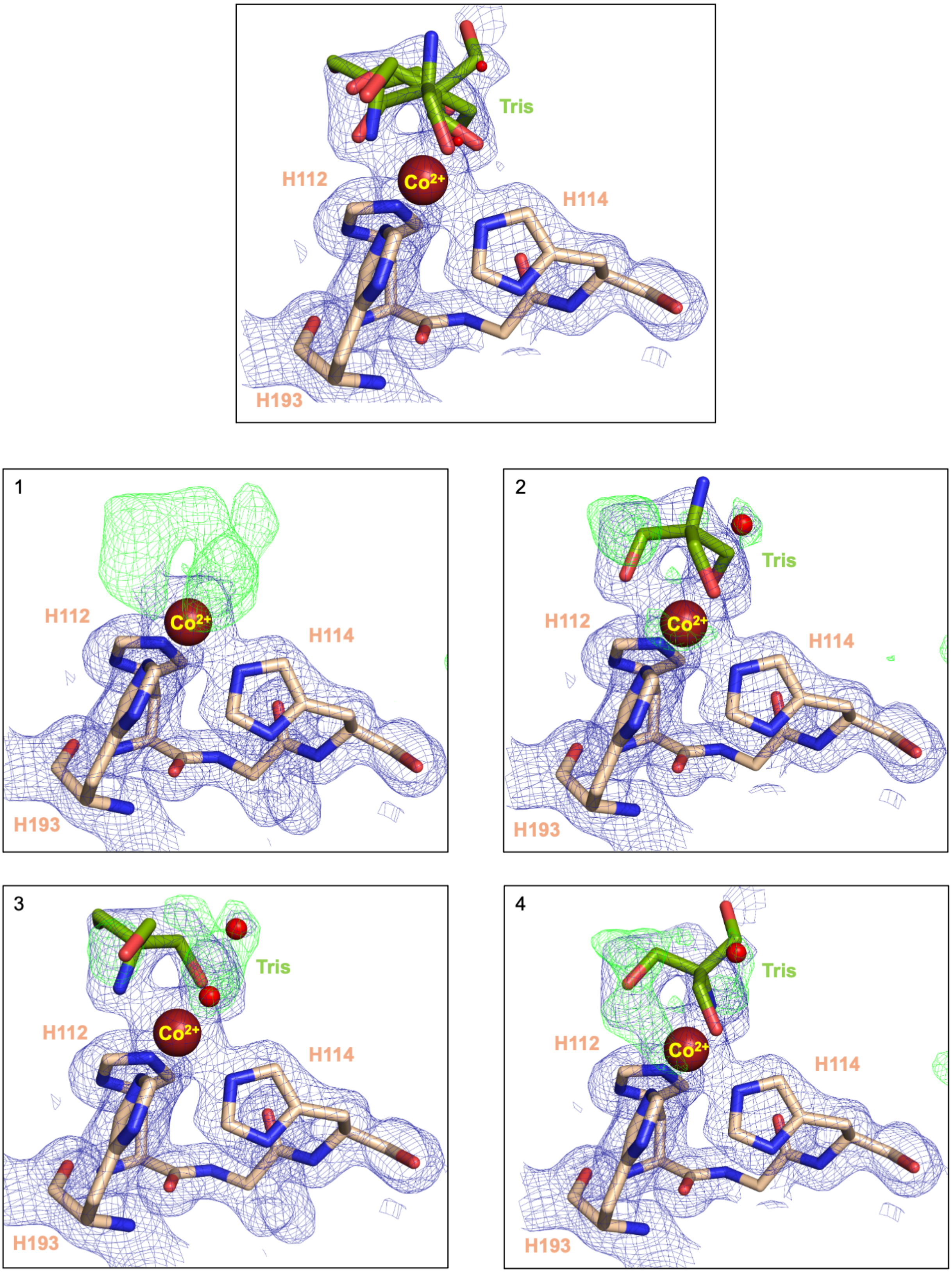

Supplementary Figure 10. Tris binds to the metal centre cobalt-incorporated ADO in complex with CP6 in multiple conformations. The crystal structure of cobalt-incorporated ADO (pink) bound to CP6 has additional electron density surrounding the metal centre, which was interpretated as Tris (green) bound in multiple conformations. 2Fo-Fc electron density is shown as a blue mesh, contoured at 1 sigma, Fo-Fc electron density is shown as a green or red mesh, contoured at 3 sigma.

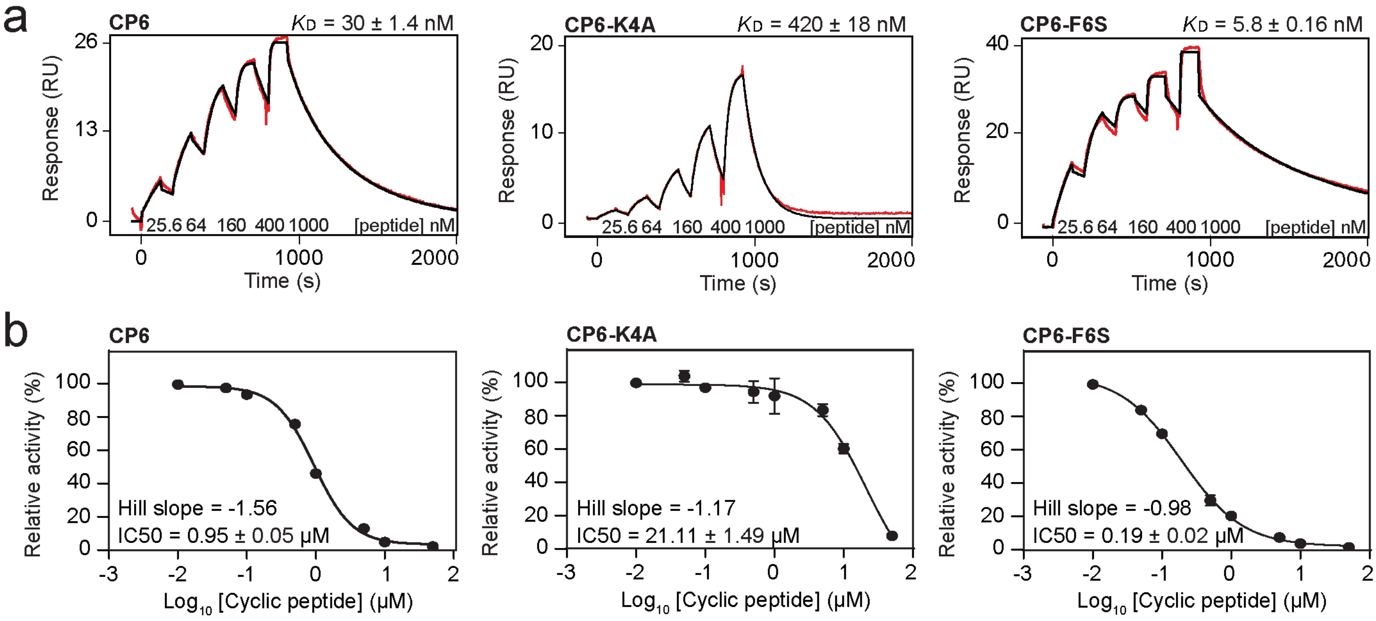

Supplementary Figure 11. Biophysical analysis of, and inhibitory responses for, CP6 substituents. a) Representative single cycle kinetic (SCK) SPR sensorgrams of CP6, CP6-K4A and CP6-F6S with ADO. The sensorgram is shown in red and the fit to the data is shown in black. The concentrations of CP used in the titration and the K_D value is shown (K_D given as the geometric mean of a minimum of three independent SPR measurements (n=3) with standard error). b) Dose-response curve with IC_{50} values for CP6, CP6-K4A and CP6-F6S. The average of three independent experiments (n=3) is shown (error bars show the standard error). Source data are provided as Source Data file.

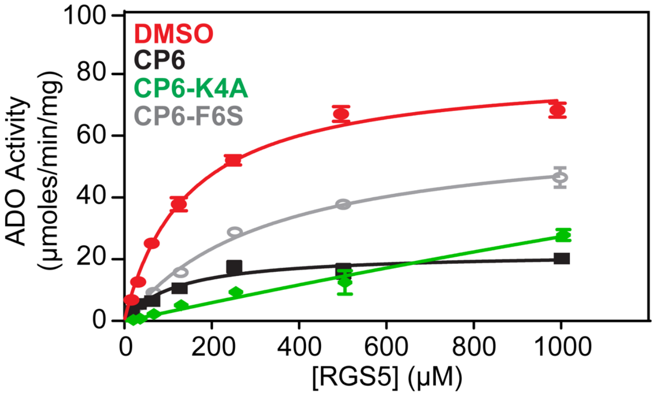

Supplementary Figure 12. Kinetic analysis of ADO in the absence and presence of CP6, CP6-K4A and CP6-F6S under aerobic conditions at 37 °C. Each CP was added at two times its IC_{50} concentration to capture changes in substrate association and turnover during partial inactivation of the enzyme (CP6 = 2 µM, CP6-K4A = 42 µM and CP6-F6S = 0.4 µM) The average of three independent experiments (n=3) is shown (error bars show the standard error). Source data are provided as Source Data file.

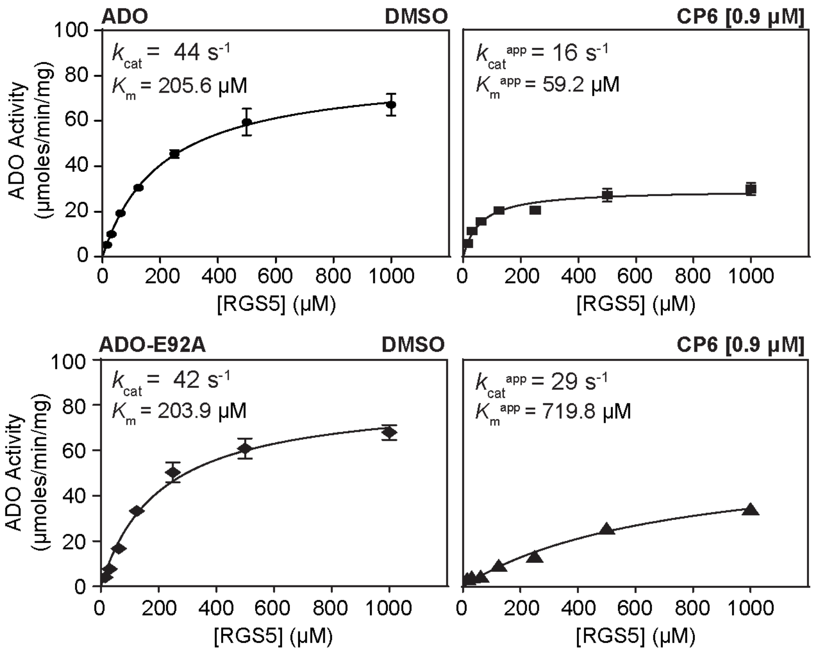

Supplementary Figure 13. **Kinetic analysis of ADO and ADO-E92A in the absence and presence of CP6.** Michaelis-Menten kinetic plots of ADO activity in the absence and presence of CP6 under aerobic conditions at 37 °C. The average of three independent experiments (n=3) is shown (error bars show the standard error). Source data are provided as Source Data file.

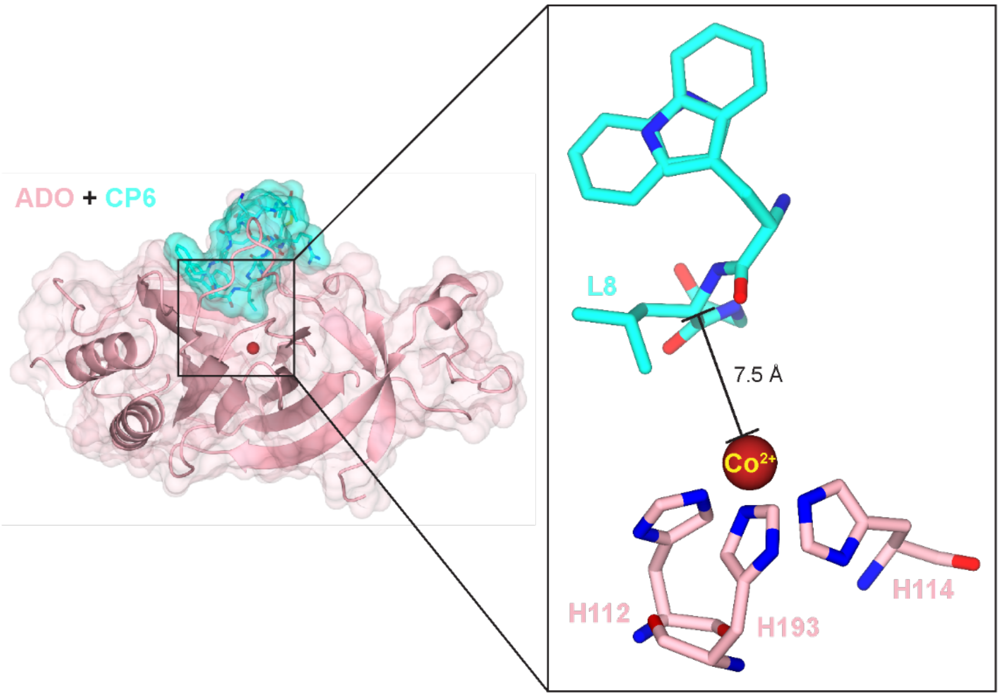

Supplementary Figure 14. The crystal structure of cobalt-incorporated ADO bound to CP6, highlighting the position of CP6-L8. CP6-L8 protrudes into the active site without contributing to binding. The α-carbon of CP-L8 is 7.5 Å away from the metal centre.

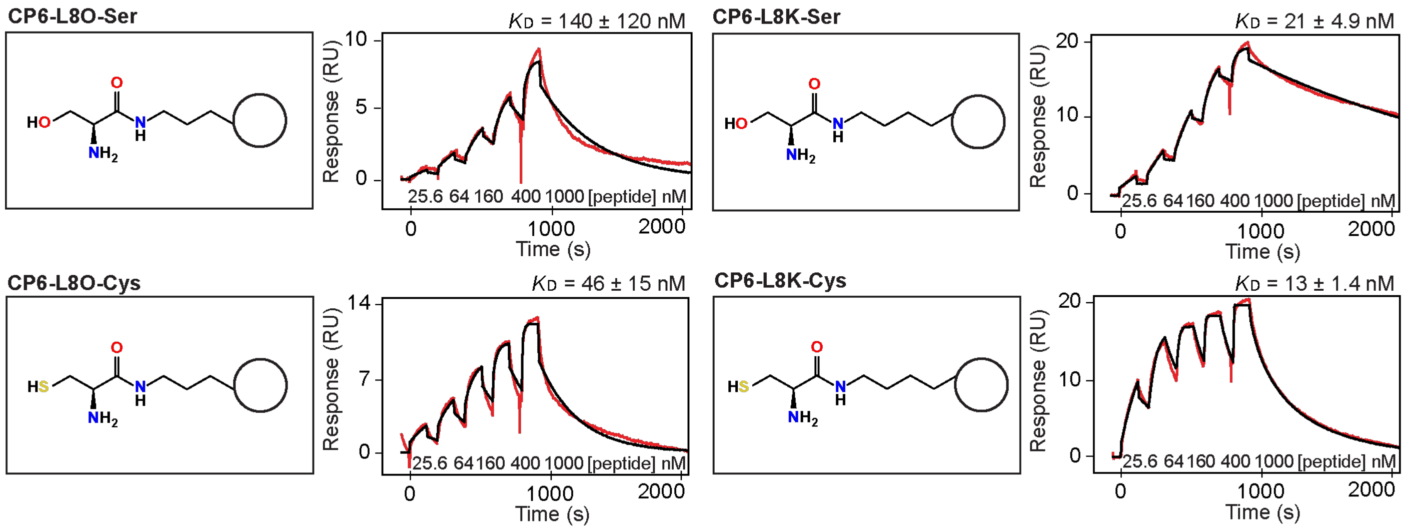

Supplementary Figure 15. Biophysical analysis of CP6 substituents bearing a substrate moiety. (*Left*) Representative chemical structures of the substrate moieties grafted onto CP6 (represented as a circle) through substitutions in CP6-L8. 'O' corresponds to ornithine. (*Right*) Single-cycle kinetic (SCK) SPR sensorgram of CP6 substituents bearing a substrate moiety with ADO. The sensorgram is shown in red and the fit to the data is shown in black. The concentrations of CP used in the titration and the K_D value are shown (K_D given as the geometric mean of a minimum of three independent SPR measurements (n=3) with standard error). Source data are provided as Source Data file.

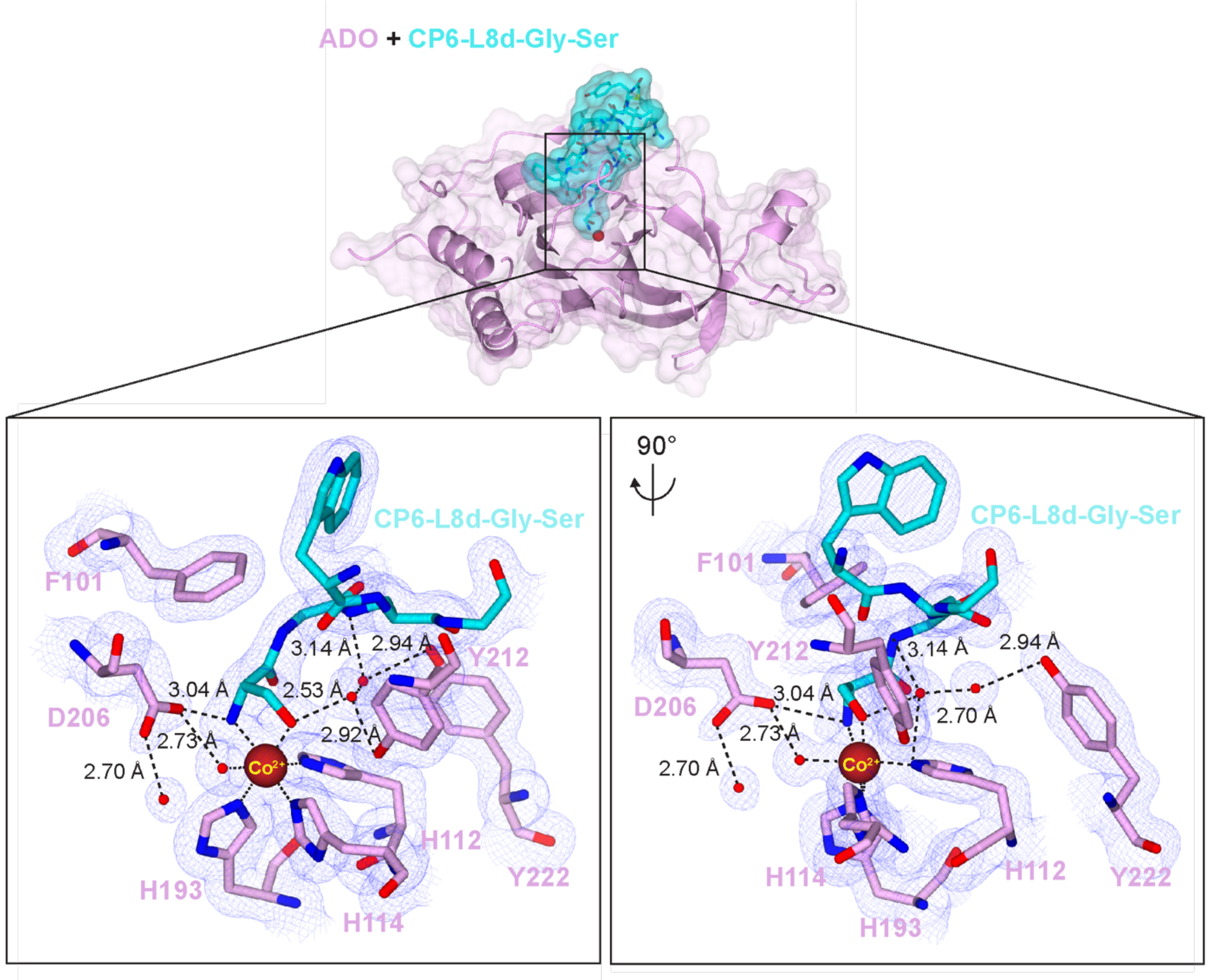

Supplementary Figure 16. The crystal structure of cobalt-incorporated ADO (pink) bound to CP6-L8d-Gly-Ser (teal) at 1.74 Å resolution. Displayed using ribbon, surface and cylinder representations, with 2Fo-Fc electron density shown as a blue mesh contoured at 1 sigma. CP6-L8d-Gly-Ser has an Nt-Ser incorporated ligand which coordinates the metal cofactor in a bidentate arrangement through ligation of the hydroxyl and amine groups. ADO-F101 forms pi stacking interaction with the substrate amide, while ADO-Y212 and -D206 form hydrogen bonds with the hydroxyl and amine of Nt-Ser, respectively. ADO-D206 interacts with the water molecule trans ADO-H112, which is the putative O_2 binding site. ADO-Y212 and -Y222 may form a water mediated hydrogen bonds with the amide substrate moiety Gly.

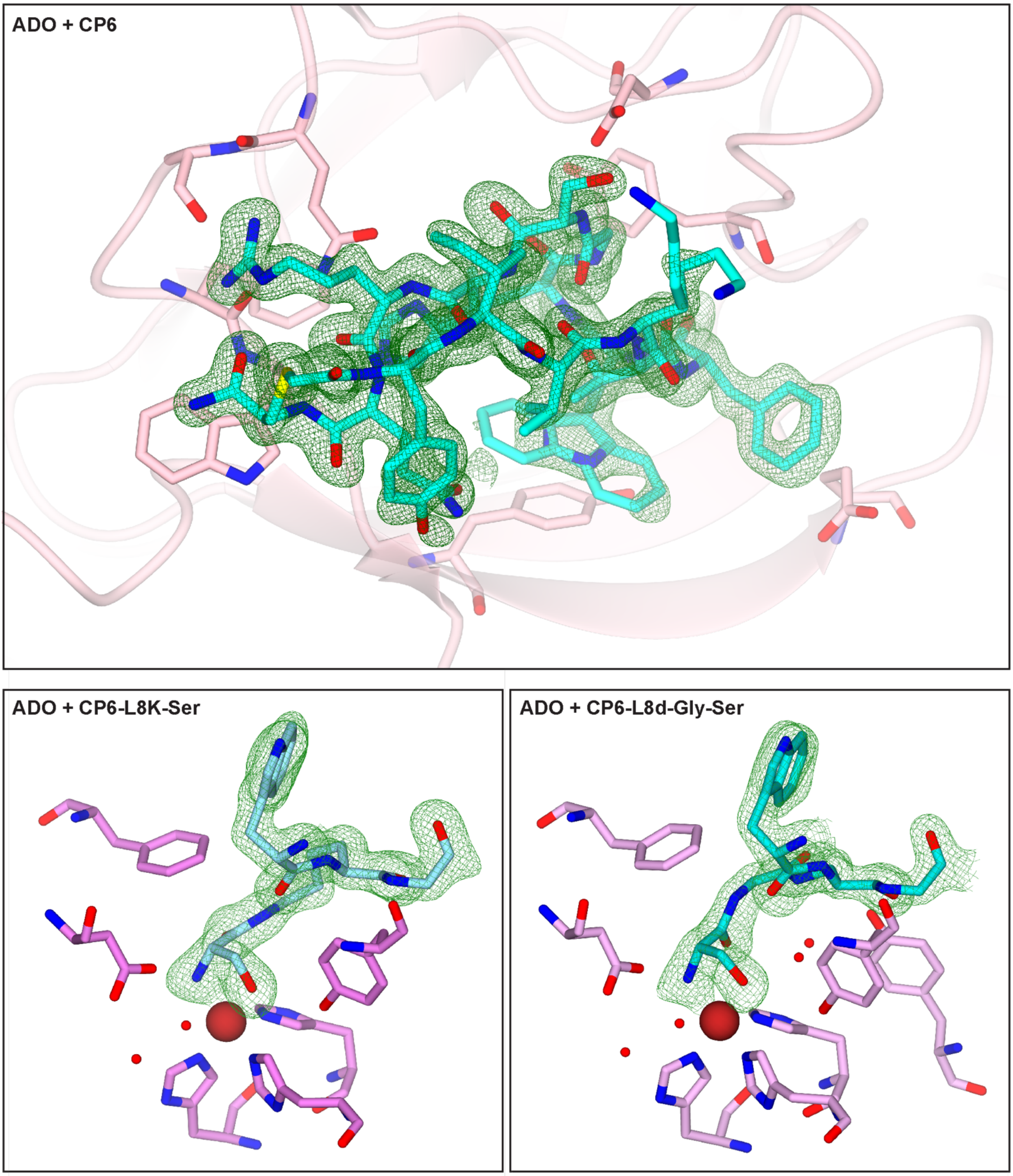

Supplementary Figure 17. The crystal structures of cobalt-incorporated ADO (pink) in complex with CP6 (top), CP6-L8K-Ser (bottom left), and CP6-L8d-Gly-Ser (bottom right), highlighting ligand fit using an omit map. Displayed using cylinder representation, with Fo-Fc electron density shown as a green mesh contoured at 3 sigma.

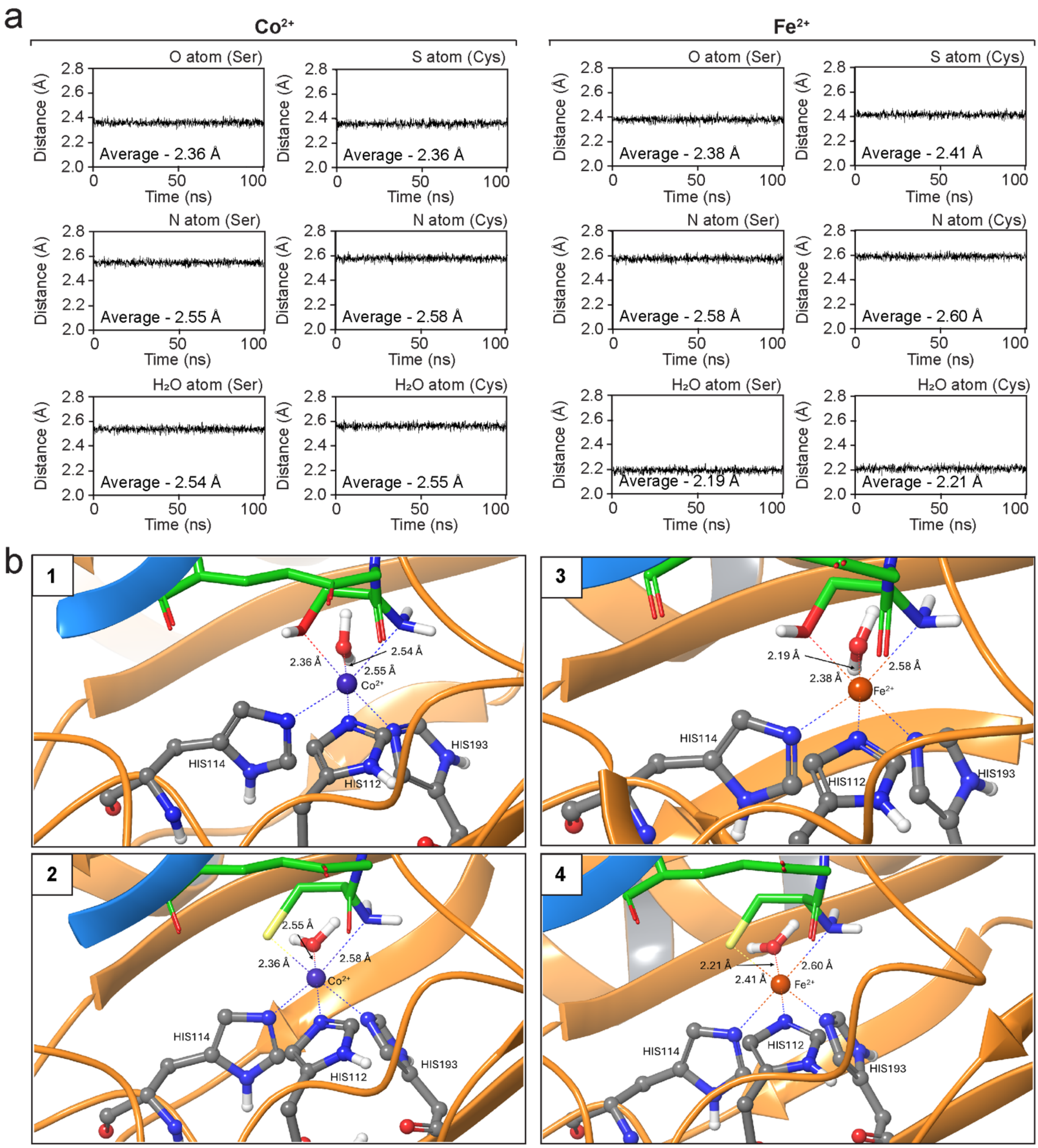

Supplementary Figure 18. Molecular dynamic (MD) simulations indicate that Nt-Ser/Cys maintains bidentate coordination with Co^{2+}/Fe^{2+}. a) Simulated movements of the ligated substrate (analogue) atoms and water molecule in the active site of ADO, using both Co^{2+} and Fe^{2+} as the metal cofactor. ADO and ligands were parameterised using the OPLS4 forcefield. The simulations were performed in triplicate (n=3) for 100 ns on each protein complex (*from left*: Ser-Co^{2+}, Cys-Co^{2+}, Ser-Fe^{2+} and Cys-Fe^{2+}) after standard energy minimization and equilibration. b) Representative models (n=3) for each simulation (Ser-Co^{2+} *(1)*, Cys-Co^{2+}*(2)*, Ser-Fe^{2+}*(3)* and Cys-Fe^{2+}*(4)*) The indicated distances (Å) on the models are given as the average distance (Å). Source data are provided as Source Data file.

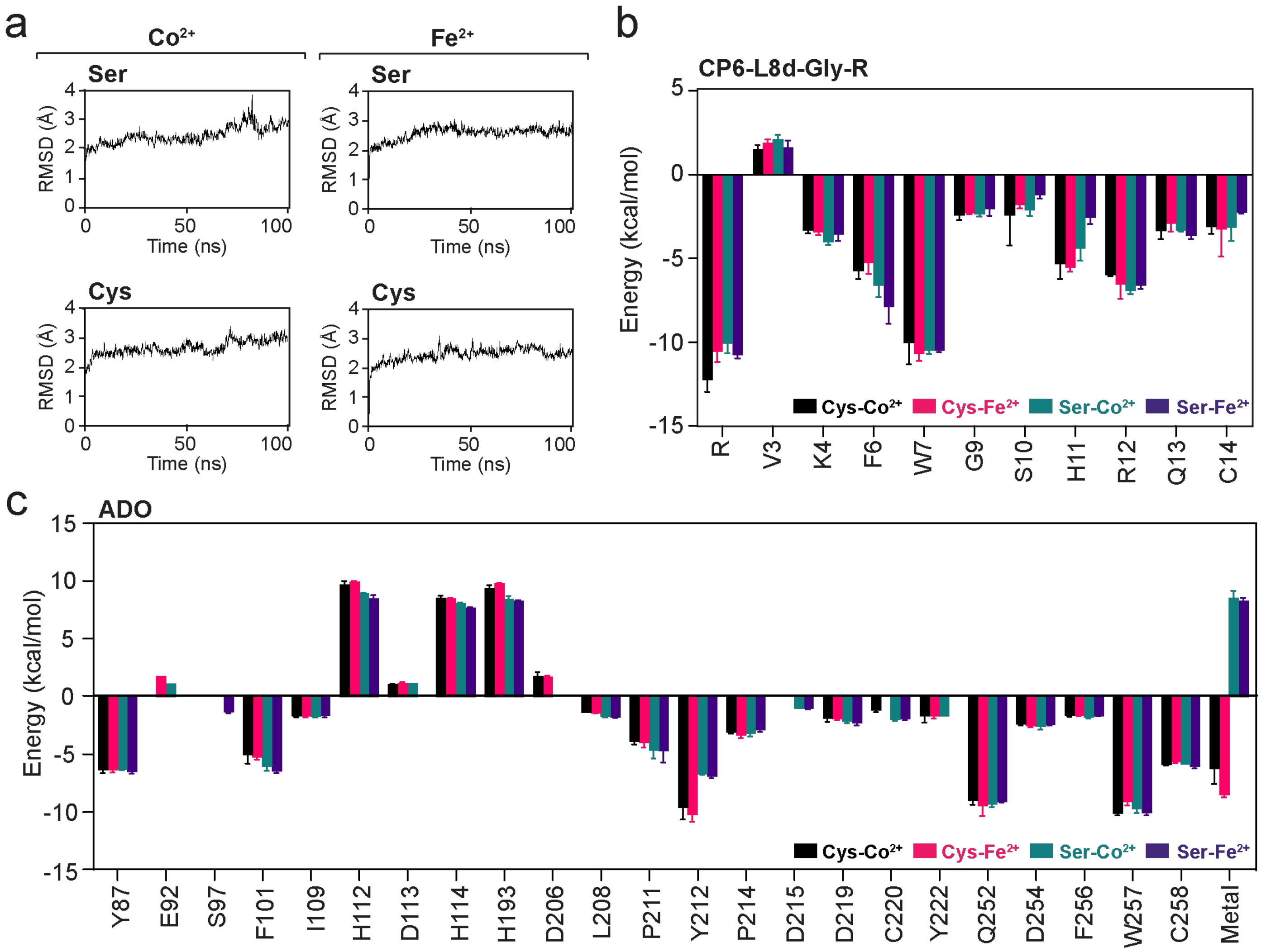

Supplementary Figure 19. Energy calculations for each interacting residues in the simulated models. a) Representative analysis of root mean square deviation (RMSD) of the ADO-ligand complex. Different combinations of pseudo-N-terminal residue and metal cofactor were explored. The simulated complex equilibriums were assessed in triplicate (n=3) for each condition for 100 ns. b) The binding energy associated with amino acid residues within CP-L8d-Gly-Ser/Cys, where R represents the pseudo-N-terminal residue (n=3, error bars show the standard deviation). Different combinations of pseudo-N-terminal residue and metal cofactor were explored. R and W7 generated the lowest binding energies. c) The binding energy associated with amino acid residues with the ADO active site (n=3, error bars show the standard deviation). Different combinations of pseudo-N-terminal residue and metal cofactor were explored. The average energy was calculated 5th frame from 50 ns to the end of the simulation based on the equilibrium trajectory of conventional MD for the interactions (Cys-Co^{2+}[*black*], Cys-Fe^{2+}[*pink*], Ser-Co^{2+}[*green*] and Ser-Fe^{2+}[*purple*]). Source data are provided as Source Data file.

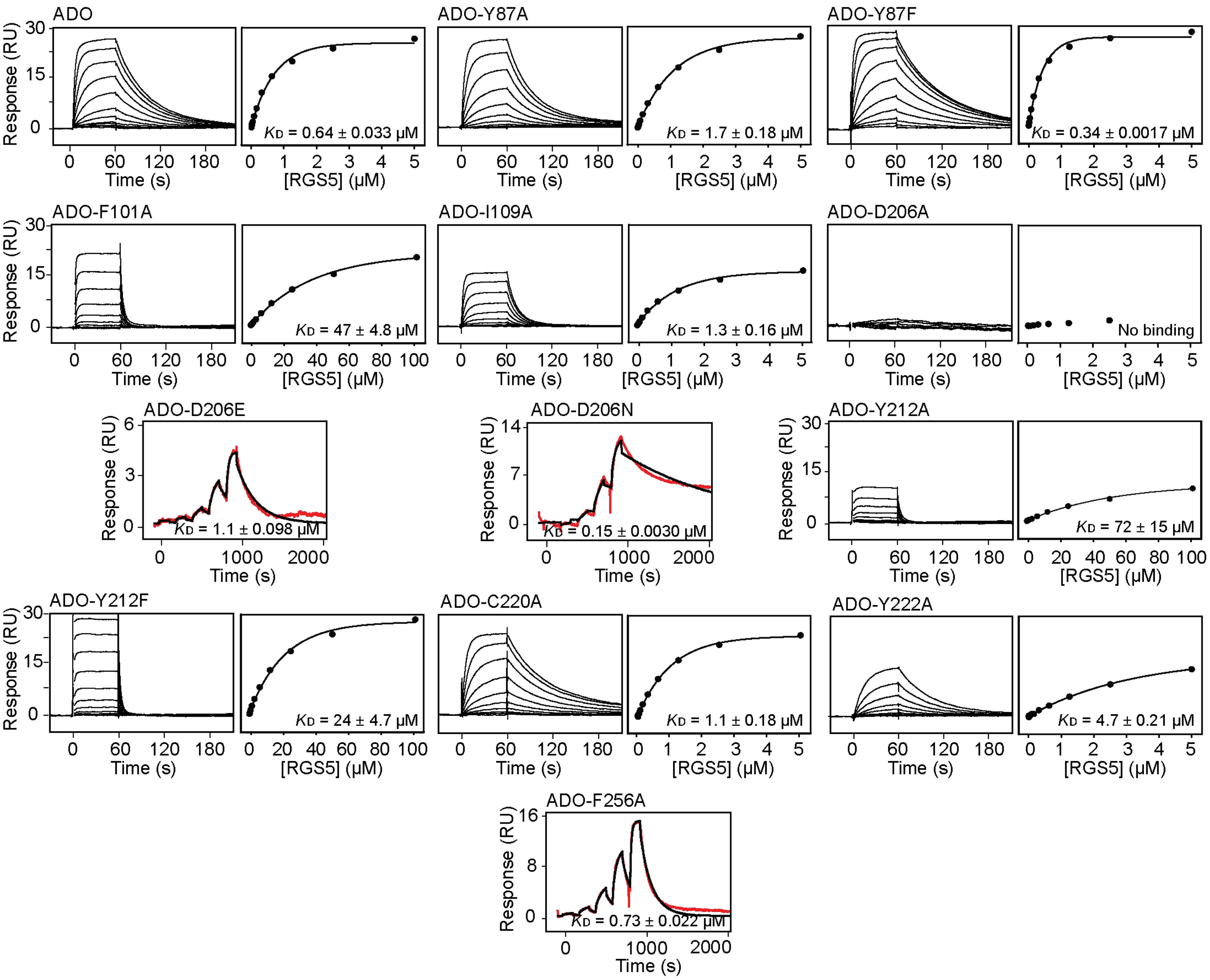

Supplementary Figure 20. SPR analysis of ADO variants with RGS5 peptide. (*Left*) Representative SPR sensorgram for the titration of $RGS5_{2\text{-}15}$ with amino acid substituted ADO. (*Right*) Fits of the equilibrium responses from the sensorgram in the left panels to a 1:1 binding model. The K_D values are shown (K_D given as the geometric mean of a minimum of three independent SPR measurements (n=3) with standard error). Interactions with significantly slow-off rate data was collected using SCK. The sensorgram is shown in red and the fit to the data is shown in black. The concentrations of $RGS5_{2\text{-}15}$ used in the titration are 25.6 nM, 64 nM, 160 nM, 400 nM, 1000 nM. The K_D value is shown (K_D given as the geometric mean of a minimum of three independent SPR measurements). Source data are provided as Source Data file.

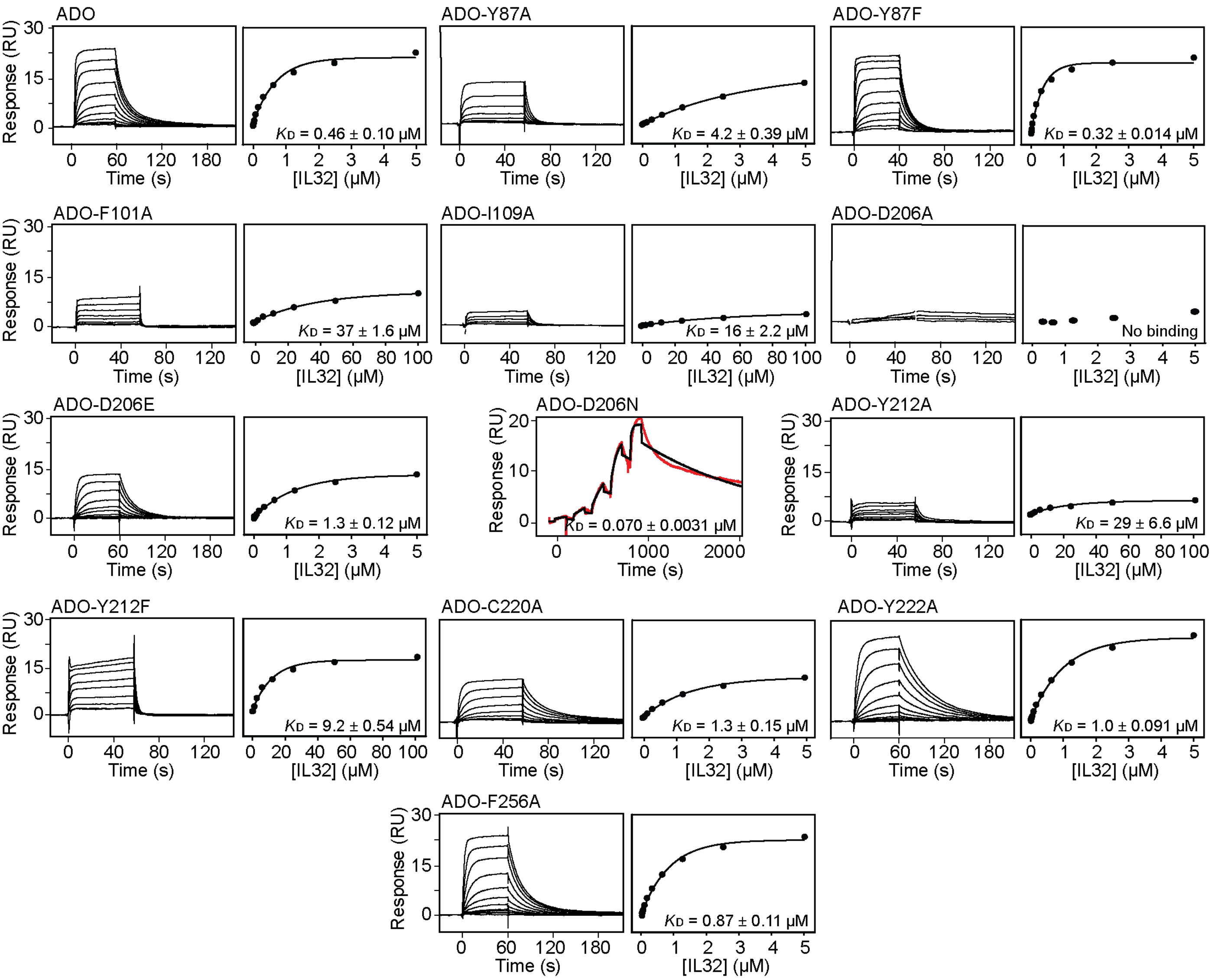

Supplementary Figure 21. SPR analysis of ADO variants with IL32 peptide. (*Left*) Representative SPR sensorgram for the titration of $IL32_{2-15}$ with amino acid substituted ADO. (*Right*) Fits of the equilibrium responses from the sensorgram in the left panels to a 1:1 binding model. The K_D values are shown (K_D given as the geometric mean of a minimum of three independent SPR measurements (n=3) with standard error). Interactions with significantly slow-off rate data was collected using SCK. The sensorgram is shown in red and the fit to the data is shown in black. The concentrations of $IL32_{2-15}$ used in the titration are 25.6 nM, 64 nM, 160 nM, 400 nM, 1000 nM. The K_D value is shown (K_D given as the geometric mean of a minimum of three independent SPR measurements). Source data are provided as Source Data file.

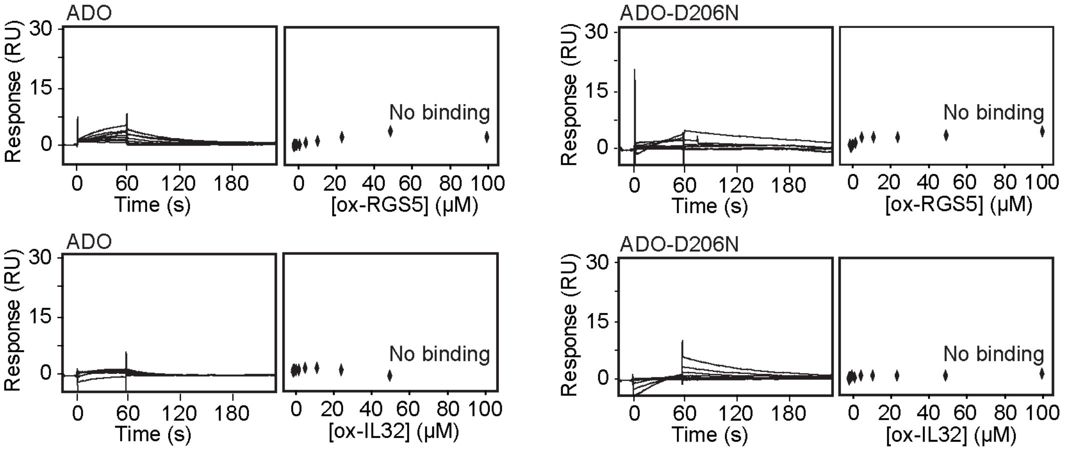

Supplementary Figure 22. SPR analysis of ADO with oxidised peptides. (*Left*) Representative SPR sensorgram for the titration of sulfinylated $RGS5_{2\text{-}15}$ and $IL32_{2\text{-}15}$ (obtained through pre-incubation with ADO) with ADO and ADO-D206N. (*Right*) The equilibrium responses from the sensorgrams are in the left panels. No binding is observed, suggesting that ADO-D206 does not promote product release.

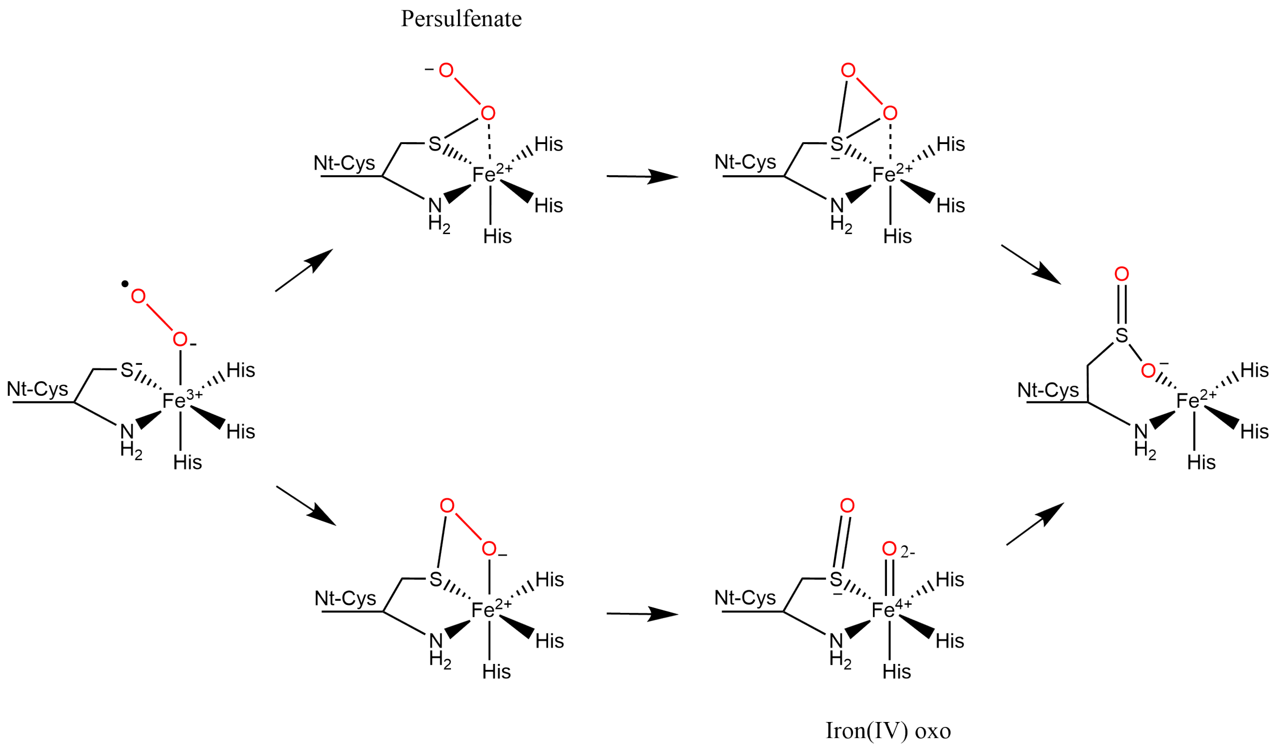

Supplementary Figure 23. Two reaction pathways proposed for ADO and other thiol dioxygenases. Initial reaction with the proximal oxygen atom results in formation of persulfenate intermediate through a concerted mechanism (top route). Initial reaction with the distal oxygen atom results in formation of an iron(IV) oxo species through a step wise mechanism (bottom route).

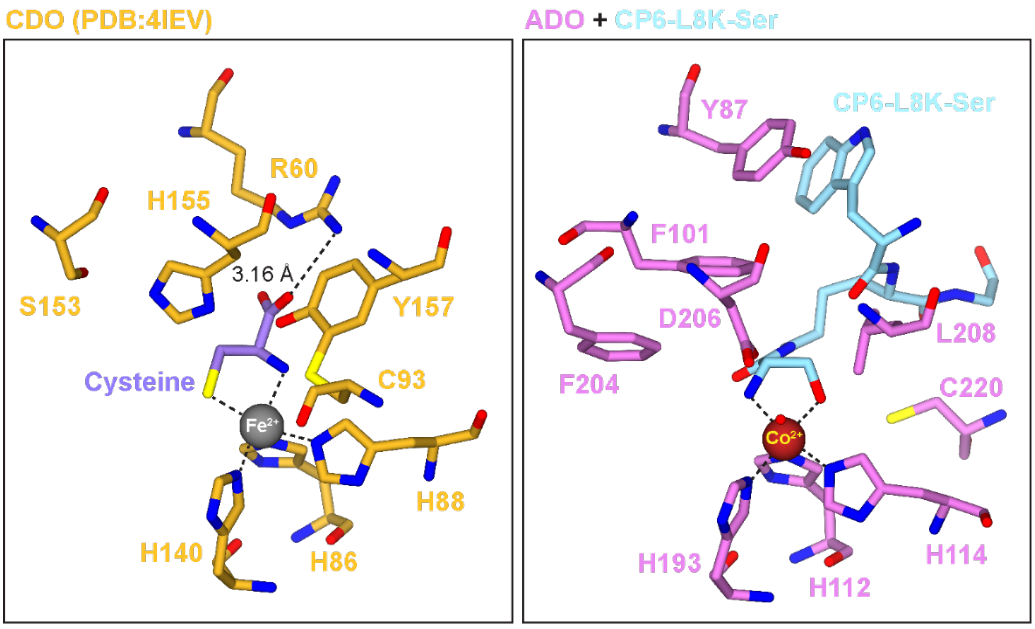

Supplementary Figure 24. Comparing the active site architecture and substrate (analogue) binding orientation of CDO (*Left*, PDB: 4IEV) and ADO in complex with CP6-L8K-Ser (Right). Both enzymes coordinate their substrate (analogue) in a bidentate arrangement, however, the orientation is reversed.

A

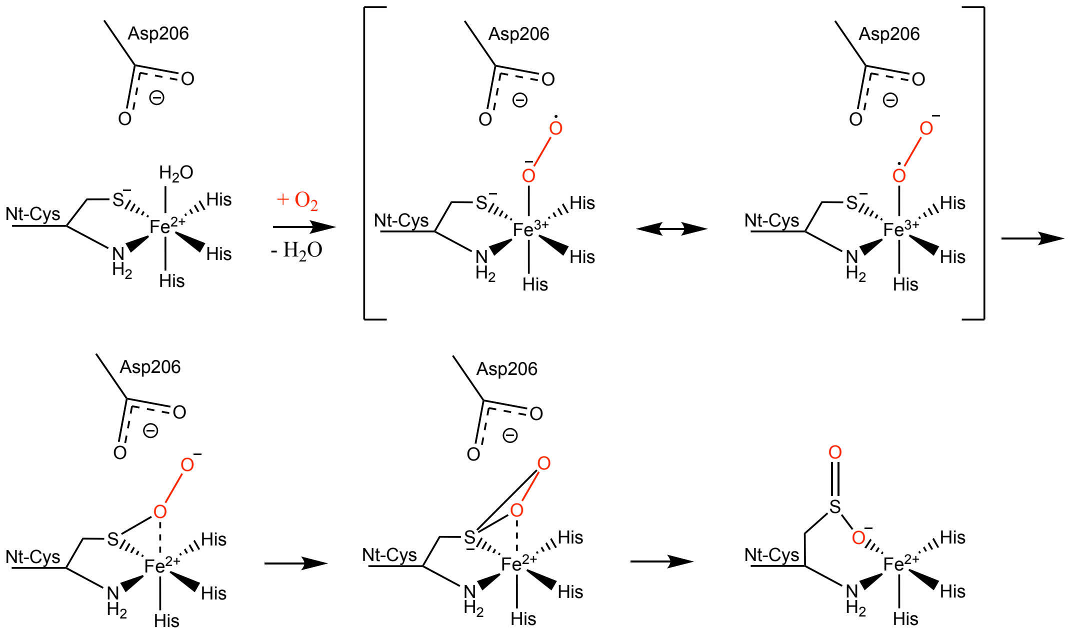

B

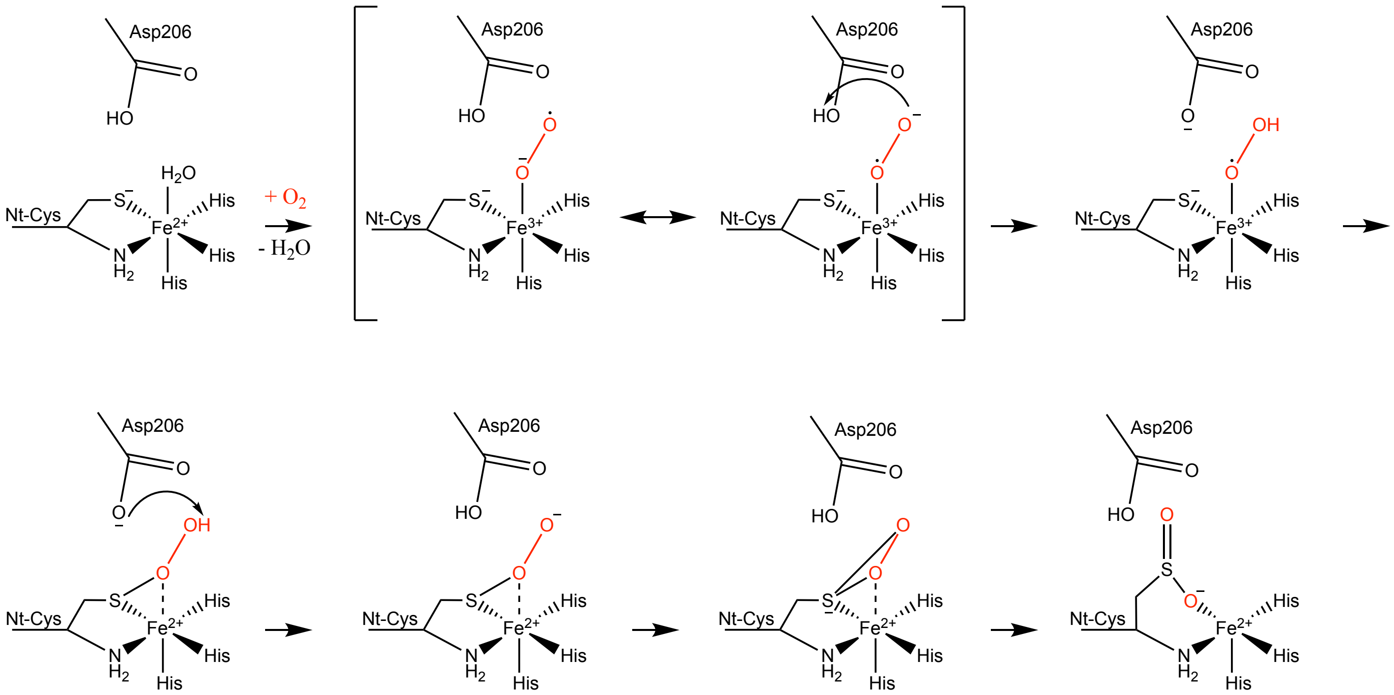

Supplementary Figure 25. A potential role for ADO-D206 during turnover part 1. ADO-D206, which sits above the N-terminal residue of the substrate, may (A) act as an orienting residue, which positions the distal oxygen away from the thiol group through charge repulsion, promoting initial reaction with the proximal oxygen, or (B) act as a directing residue that transiently protonates the distal oxygen, increasing the electrophilicity and reactivity of the proximal oxygen.

A

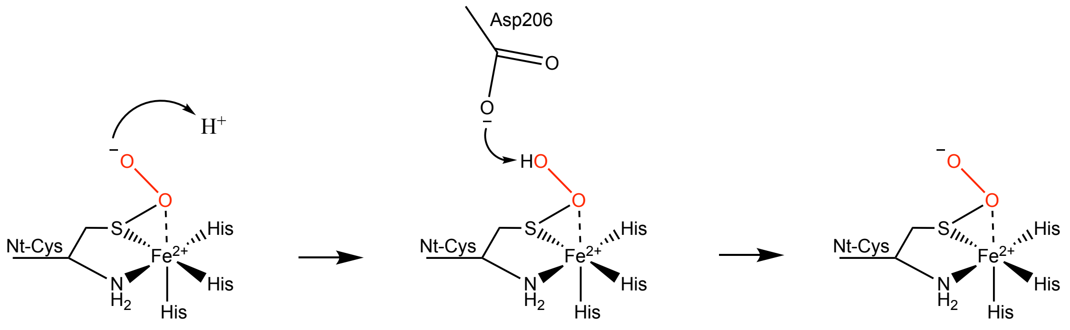

B

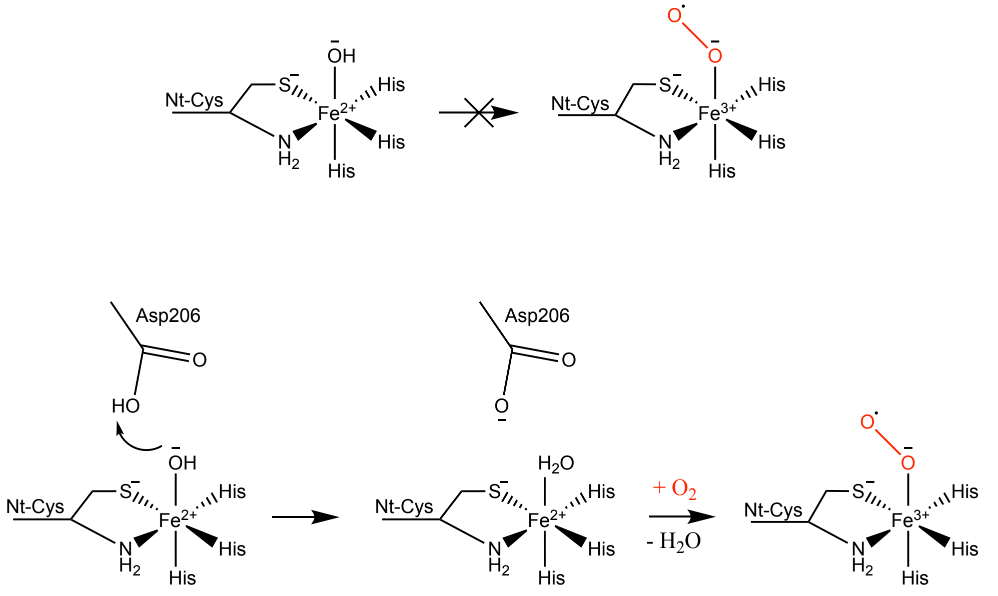

Supplementary Figure 26. A potential role for ADO-D206 during turnover part 2. ADO-D206, which sits above the N-terminal residue of the substrate, may (A) act as a stabilizing residue, which prevents protonation of a reactive oxygen intermediate, stopping the reaction from stalling, or (B) maintain the protonation state of the water occupying the O_2 binding site, facilitating exchange with O_2.

Supplementary tables

Supplementary Table 1. The (apparent) kinetic parameters of ADO in the presence and absence of different CPs. Values were calculated using data presented in Supplementary Figure 7.

Treatments	$V_{max}^{(app)}$ (µmoles/min/mg)	$k_{cat}^{(app)}$ (s^{-1})	$K_m^{(app)}$ (µM)	Catalytic effciency ($s^{-1}M^{-1}$)
DMSO	85.76	45.62	178.4	2.6×10^5
CP1	92.82	49.37	467.7	1.1×10^5
CP5	88.78	47.22	250.7	1.9×10^5
CP6	29.58	15.73	59.2	2.7×10^5
CP8	19.92	10.69	21.6	4.9×10^5

Supplementary Table 2. A summary of intramolecular interactions observed for CP6.

Interaction	Residue 1	Residue 2	Distance (Å)
1	Y1 carbonyl O	Q13 amine N	2.90
2	Y1 sidechain O	Q13 sidechain N	2.89
3	V3 amine N	H11 carbonyl O	2.90
4	V3 carbonyl O	H11 amine N	2.80
5	K4 amine N	S10 sidechain O	2.89
6	T5 amine N	G9 carbonyl O	2.92
7	T5 sidechain O	L8 amine N	2.92
8		G9 carbonyl O	2.85
9	R12 sidechain N	C14 carbonyl O	2.79
10			2.87

Supplementary Table 3. A summary of intermolecular interactions observed between ADO and CP6.

Box No.	ADO residue	CP6 residue	Distance (Å)	Interaction
1	A253 carbonyl O	R12 sidechain N	3.08	H bond
	F256 carbonyl O	R12 sidechain N	3.07	H bond
2	Q252 sidechain N	R12 carbonyl O	2.92	H bond
	Q252 sidechain O	R12 amine N	2.96	H bond
3	D219 sidechain O	S10 sidechain O	2.75	H bond
	D219 sidechain O	S10 amine N	2.71	H bond
4	Y212 amine N	F6 carbonyl O	3.08	H bond
5	Y87 sidechain	W7 sidechain	-	pi (π) stacking
6	W257 sidechain	C14 sidechain	-	pi (π) stacking

Supplementary Table 4. Biophysical, inhibition and kinetic data for ADO with and without CP6 and its substituents (added at its IC_{50} concentration). Values were calculated using data presented in Supplementary Figure 11 and Figure 2C.

Conditions	K_D (nM)	IC50 (μM)	$V_{max}^{(app)}$ (μmoles/min/mg)	$k_{cat}^{(app)}$ (s^{-1})	$K_m^{(app)}$ (μM)	Catalytic effciency ($s^{-1}M^{-1}$)
DMSO	-	-	82.15	43.70	205.6	2.1×10^5
CP6	30 ± 1.4	0.95 ± 0.05	29.58	15.73	59.2	2.7×10^5
CP6-K4A	420 ± 18	21.11 ± 1.49	56.60	30.11	388.6	7.7×10^4
CP6-F6S	5.8 ± 0.16	0.19 ± 0.02	71.92	38.25	181.0	2.1×10^5

Supplementary Table 5. Kinetic data for ADO with and without CP6 and its substituents (added at two times its IC_{50} concentration). Values were calculated using data presented in Supplementary Figure 12.

Conditions	$V_{max}^{(app)}$ (μmoles/min/mg)	$k_{cat}^{(app)}$ (s^{-1})	$K_m^{(app)}$ (μM)	Catalytic effciency ($s^{-1}M^{-1}$)
DMSO	81.05	43.11	148.4	2.9×10^5
CP6	21.90	11.65	119.8	9.7×10^4
CP6-K4A	Undefined	Undefined	Undefined	Undefined
CP6-F6S	63.80	33.94	329.7	1.0×10^5

Supplementary Table 6. The (apparent) kinetic parameters of ADO and ADO-E92A in the presence and absence of CP6. Values were calculated using data presented in Supplementary Figure 13.

Enzymes	Treatments	$V_{max}^{(app)}$ (μmoles/min/mg)	$k_{cat}^{(app)}$ (s^{-1})	$K_m^{(app)}$ (μM)	Catalytic effciency ($s^{-1}M^{-1}$)
ADO	DMSO	82.15	43.70	205.6	2.1×10^5
	CP6	29.58	15.73	59.2	2.7×10^4
ADO-E92	DMSO	84.27	41.70	203.9	2.0×10^5
	CP6	59.29	29.34	719.8	4.1×10^4

Supplementary Table 7. Equilibrium dissociation constants for the binding of RGS5 and IL32 peptides to ADO and its variants. Values were calculated using data presented in Supplementary Figures 19 and 20.

ADO	$RGS5_{2\text{-}15}$		$IL32_{2\text{-}15}$	
	K_D (μM)	Relative K_D (fold difference)	K_D (μM)	Relative K_D (fold difference)
Wild type	0.64 ± 0.033	1.00	0.46 ± 0.10	1.00
Y87A	1.7 ± 0.18	2.58 ± 0.28	4.2 ± 0.39	9.10 ± 0.85
Y87F	0.34 ± 0.0017	1.89 ± 0.01	0.32 ± 0.014	1.43 ± 0.07
F101A	47 ± 4.8	73.36 ± 7.58	37 ± 1.6	80.47 ± 3.49
I109A	1.3 ± 0.16	1.96 ± 0.24	16 ± 2.2	34.54 ± 4.83
D206A	No binding	-	No binding	-
D206N	0.15 ± 0.0030	4.21 ± 0.08	0.070 ± 0.0031	6.56 ± 0.29
D206E	1.1 ± 0.098	1.69 ± 0.15	1.3 ± 0.12	2.85 ± 0.26
Y212A	72 ± 15	113.11 ± 23.17	29 ± 6.6	63.35 ± 14.38
Y212F	24 ± 4.7	37.88 ± 7.27	9.2 ± 0.54	20.16 ± 1.18
C220A	1.1 ± 0.18	1.75 ± 0.27	1.3 ± 0.15	2.94 ± 0.32
Y222A	4.7 ± 0.21	7.29 ± 0.33	1.0 ± 0.091	2.22 ± 0.20
F256A	0.73 ± 0.022	1.14 ± 0.03	0.87 ± 0.11	1.90 ± 0.25

Supplementary Table 8. Specific and relative activity data for ADO and its variants with RGS5 and IL32 peptide. Values were calculated using data presented in Figure 4C.

ADO	$RGS5_{2-15}$		$IL32_{2-15}$	
	ADO activity (μmoles/min/mg)	Relative activity (%)	ADO activity (μmoles/min/mg)	Relative activity (%)
Wild type	71.04 ± 3.00	100	41.08 ± 2.04	100
Y87A	25.41 ± 1.01	36.01 ± 1.44	28.25 ± 0.80	68.77 ± 1.95
Y87F	23.56 ± 2.96	33.39 ± 4.20	26.65 ± 2.22	64.88 ± 5.40
F101A	5.75 ± 0.57	8.04 ± 0.79	12.43 ± 1.93	30.25 ± 4.69
I109A	22.69 ± 2.34	31.73 ± 3.27	24.58 ± 3.22	59.83 ± 7.83
D206A	0.66 ± 0.06	0.92 ± 0.08	0.23 ± 0.06	0.56 ± 0.14
D206N	1.28 ± 0.07	1.79 ± 0.10	0.82 ± 0.05	2.00 ± 0.11
D206E	9.19 ± 0.83	12.85 ± 1.16	7.99 ± 1.10	19.44 ± 2.68
Y212A	7.07 ± 0.13	9.89 ± 0.18	26.44 ± 2.44	64.36 ± 5.93
Y212F	38.90 ± 1.85	54.40 ± 2.58	32.33 ± 2.88	78.45 ± 7.01
C220A	34.05 ± 2.69	48.25 ± 3.81	18.27 ± 0.33	44.47 ± 0.81
Y222A	47.45 ± 2.05	66.36 ± 2.87	58.45 ± 4.68	142.26 ± 11.40
F256A	45.98 ± 3.64	64.30 ± 5.09	53.07 ± 4.63	129.18 ± 11.28

Supplementary Table 9. Data collection and refinement crystallography statistics for ADO in complex with each CP6 and its substituents.

	ADO CP6<br>PDB: 9DXU	ADO CP6-L8K-Ser<br>PDB: 9DXV	ADO CP6-L8d-Gly-Ser<br>PDB: 9DXB
**Data collection**			
Space group	P 2 21 21	P 2 21 21	P 2 21 21
Cell dimensions			
a, *b*, *c* (Å)	36.11, 71.61, 108.87	35.94, 71.59, 108.53	36.02, 71.88, 108.89
α, β, γ (°)	90, 90, 90	90, 90, 90	90, 90, 90
Resolution (Å)	43.34 – 1.74 (1.78 – 1.74)	43.24 – 1.60 (1.62 – 1.60)	43.40 – 1.74 (1.77 – 1.74)
R_{sym} or R_{merge}	0.098 (1.29)	0.062 (1.17)	0.055 (0.79)
$I / \sigma I$	12.6 (1.4)	15.7 (1.3)	18.1 (2.0)
Completeness (%)	99.9 (97.7)	99.3 (86.2)	99.8 (96.0)
Redundancy	13.2 (13.1)	13.2 (12.2)	13.0 (12.4)
**Refinement**			
Resolution (Å)	1.74	1.60	1.74
No. reflections	29515	37801	29967
R_{work} / R_{free}	0.175/0.215	0.171/0.192	0.177/0.219
No. atoms			
Protein	1923	1890	1913
Peptide	143	133	134
Ligand/ion	76	77	32
Metal	1	1	1
Water	243	221	200
B-factors			
Protein	28.82	32.99	33.91
Peptide	25.58	33.03	32.69
Ligand/ion	41.88	61.46	50.18
Metal	17.26	20.95	19.10
Water	41.73	44.24	45.08
R.m.s. deviations			
Bond lengths (Å)	0.38	0.39	0.36
Bond angles (°)	0.65	0.69	0.67

Supplementary methods

Fmoc-Solid-Phase Peptide Synthesis (SPPS)

General Materials and Methods

Peptide grade *N,N*-dimethylformamide (DMF) for peptide synthesis was purchased from Labscan. Wash-grade DMF was purchased from Ajax. Fmoc-protected amino acids, coupling reagents and resins used for solid-phase synthesis were purchased from the following suppliers: Bachem, GL Biochem, Sigma-Aldrich, Novabiochem, and AK Scientific.

Analytical Liquid Chromatography Mass Spectrometry (LCMS)

LC-MS analysis was performed on a Shimadzu UPLC-MS equipped with an SPD-M30A diode array detector and a Shimadzu 2020 (ESI) mass spectrometer functioning in positive mode. Analytical samples were analysed using a Waters Acquity UPLC BEH C8 column (1.7 µm 2.1 × 50 mm) at a flow rate of 0.6 mL/min using a mobile phase of 0.1% formic acid in H_2O (Solvent A) and 0.1% formic acid in MeCN (Solvent B) and a linear gradient with increasing %B over 3, 5 or 8 min on the UPLC-MS system.

Reversed-Phase HPLC

Analytical reversed-phase HPLC was carried out using a Waters Alliance e2695 HPLC system equipped with a 2998 PDA detector (λ = 210–400 nm) and a column heater (Alliance series) at 50 °C. Peptides were analysed using the Waters SunFire C18 column (5 µm 2.1 × 150 mm) at a flow rate of 0.7 mL/min on the HPLC system. Instruments used mobile phases composed of 0.1% TFA in H_2O (Solvent A) and 0.1% TFA in MeCN (Solvent B) on a linear gradient of 0% to 50% B over 30 min. GraphPad Prism was used to process the raw chromatograms, such that zero minutes represents the start of the gradient.

Preparative reversed-phase HPLC purification was performed using a Waters 600E Multisolvent Delivery System with a Rheodyne 7725i Injection valve (5 mL loading loop) and Waters 500 pump with a Waters 490E programmable wavelength detector operating at 214 and 280 nm. HPLC was performed using a Waters Sunfire C18 column (5 µm, 10 × 250 mm) at a flow rate of 14 mL/min unless otherwise specified. A mobile phase of 0.1% TFA in H_2O (Solvent A) and 0.1% TFA in MeCN (Solvent B) using the linear gradients specified. After lyophilisation, homogeneous peptides were obtained as trifluoroacetate salts.

General procedure A: Automated Peptide Synthesis (SYRO I peptide synthesiser)

Rink Amide resin (50 µmol, ~0.6 mmol/g) in polypropylene reaction vessels was Fmoc-deprotected with 40 vol.% piperidine in DMF (800 µL) for 3 min with intermittent shaking, drained by vacuum, and treated with 20 vol.% piperidine in DMF (800 µL) for 10 min, drained, and washed with DMF (4 × 1.2 mL). The resin was then treated with a solution of Fmoc-X_{aa}-OH (4 eq.) and Oxyma (4.4 eq.) in DMF (400 µL), followed by the addition of a 1 wt.% solution of 1,3-diisopropyl-2-thiourea in DMF (400 µL), and a solution of *N,N′*-diisopropylcarbodiimide (4 eq.) in DMF (400 µL). Coupling reactions were conducted at 75 °C using the heating block for 15 min and intermittently shaken. In case of Fmoc-Cys(Trt)-OH and Fmoc-His(Trt)-OH residues, the coupling was carried out at 50 °C for 30 min with intermittent shaking. The resin vessel was then vacuum drained and washed with DMF (4 × 1.2 mL). A capping step was performed with a solution of 5 vol.% Ac_2O and 10 vol.% *i*-Pr_2NEt in DMF (800 µL) for 6 min with intermittent shaking at room temperature following each coupling reaction. The resin vessel was then vacuum drained and washed with DMF (4 × 1.2 mL). Iterative cycles of this process were repeated until complete peptide elongation was achieved followed by a final Fmoc-deprotection cycle. The chloroacetyl moiety was then installed through treatment of the resin with chloroacetic acid (4 eq.) and Oxyma (4.4 eq.) in DMF (400 µL), followed by the addition of a 1 wt.% solution of 1,3-diisopropyl-2-thiourea in DMF (400 µL), and a solution of *N,N′*-diisopropylcarbodiimide (4 eq.) in DMF (400 µL). The chloroacetylation reaction was incubated at 75 °C for 15 min with intermittent shaking, after which the resin was drained, and washed with DMF (4 × 5 mL) and CH_2Cl_2 (5 × 5 mL).

General procedure B: *En bloc* DDE Removal

The resin was treated with 5 vol.% hydrazine in DMF (5 mL) for 2 × 30 min. Removal of the DDE group was confirmed by test cleavage of the resin. The resin was then washed with DMF (5 × 5 mL), followed by CH_2Cl_2 (5 × 5 mL) and DMF (5 × 5 mL).

General procedure C: Standard Preparative Cleavage

The resin (50 µmol) was thoroughly washed with CH_2Cl_2 (10 × 5 mL) before being treated with 90:5:5 v/v/v TFA:tri*iso*propylsilane:H_2O and shaken at room temperature for 2 h. The resin was filtered and the filtrate was concentrated under a stream of nitrogen before precipitating with diethyl ether (50 mL). The peptide was pelleted by centrifugation (4 min, 2 ºC, at 5800 *g*).

General procedure D: Cyclisation of Chloroacetyl-Cysteinyl Peptides

Crude peptide (50 µmol) was dissolved in a solution of DMSO/*i*-Pr_2NEt (~4 mL, 9:1, v/v). This reaction mixture was then heated to 60 °C for 1 h, or until complete macrocyclisation was observed following UPLC-MS analysis.

General procedure E: Cyclisation of Chloroacetyl-Cysteinyl Peptides in H_2O:MeCN

Crude linear peptide was first purified by RP-HPLC. HPLC fractions containing the peptide were then basified through an addition of *i*-Pr_2NEt (0.25 vol.%) and the solution was incubated at room temperature for 1 h. Following confirmation of complete cyclisation by LCMS, the fractions were lyophilised to obtain the crude cyclic peptide.

CP1

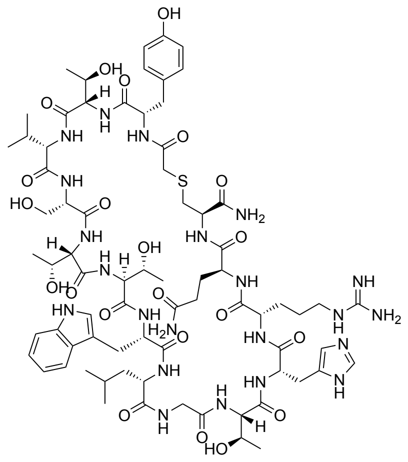

The linear peptide (50 µmol) was synthesized according to general procedure A before being cleaved from resin by general procedure C. The crude linear peptide was then purified by preparative RP-HPLC (0 vol.% CH_3CN + 0.1 vol.% TFA for 5 min, then 0-60 vol.% CH_3CN + 0.1 vol.% TFA over 80 min, 38 mL/min, XBridge® C18, 300 Å, 30 x 150 mm). The peptide was then cyclised by general procedure E before purification of the cyclic peptide by preparative HPLC (0 vol.% CH_3CN + 0.1 vol.% TFA for 10 min, then 0-60 vol.% CH_3CN + 0.1 vol.% TFA over 40 min, 38 mL/min, XBridge® C18, 300 Å, 30 x 150 mm) to afford ADO-L-01 as a white solid (11.13 mg, 13%). **A)** Analytical HPLC: $R_{t\ 214nm}$: 16.7 min. (1 to 60 vol.% CH_3CN+ 0.1 vol.% TFA over 30 min). **B)** MS (+ESI): m/z = 1692.25 $[M+H]^+$, 846.7 $[M+2H]^2$.

A) Analytical HPLC Trace (λ= 214 nm)

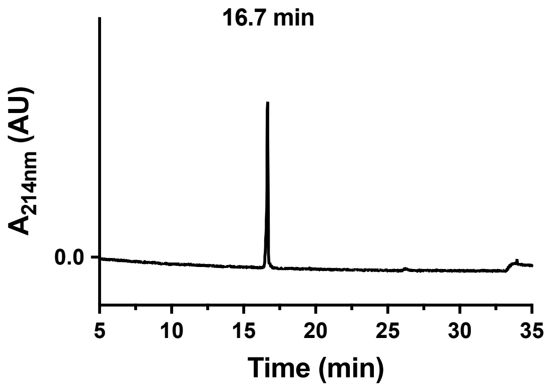

B) ESI MS (+)

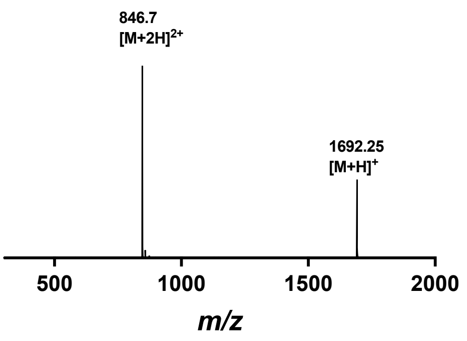

CP2

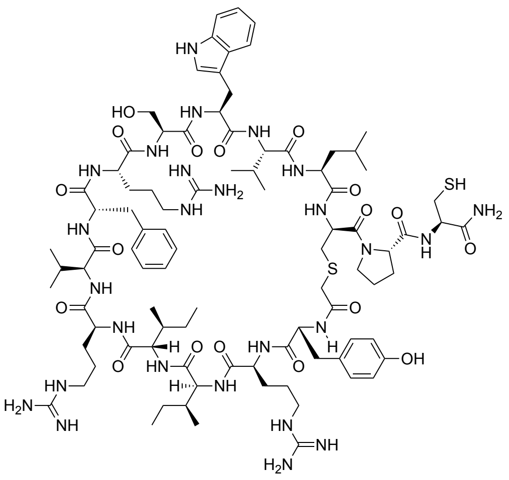

The peptide (50 µmol) was synthesized by general procedure A installing Cys16 as the acid stable Fmoc-Cys(Acm)-OH building block before being cleaved from resin by general procedure C. The crude linear peptide was then purified by preparative RP-HPLC (0 vol.% CH_3CN + 0.1 vol.% TFA for 5 min, then 0-60 vol.% CH_3CN + 0.1 vol.% TFA over 80 min, 38 mL/min, XBridge® C18, 300 Å, 30 x 150 mm). Fractions containing the linear peptide were combined and lyophilised before being redissolved in MeCN:H2O (5 mL) and cyclised through addition of *i*-Pr_2NEt (2.5 vol.%) for 1 h at rt. AgOAc (66 mg, 400 µmol) was then added and the solution shaken for 16 h at rt before addition of dithiothreitol (90 mg, 600 µmol) causing generation of a precipitate that was removed by filtration and the filtrate lyophilized. The product was purified by preparative HPLC (0 vol.% CH_3CN + 0.1 vol.% TFA for 10 min, then 0-60 vol.% CH_3CN + 0.1 vol.% TFA over 40 min, 38 mL/min, XBridge® C18, 300 Å, 30 x 150 mm) to afford the pure peptide as a white amorphous solid (0.5 mg, 0.5%). **A)** Analytical HPLC $R_{t\,214nm}$: 21.7 min. (1 to 60 vol.% CH_3CN+ 0.1 %vol TFA over 30 min). **B)** MS (+ESI): m/z = 1950.75 $[M+H]^+$, 975.95 $[M+2H]^{2+}$, 651.00 $[M+3H]^{3+}$.

A) Analytical HPLC Trace (λ= 214 nm)

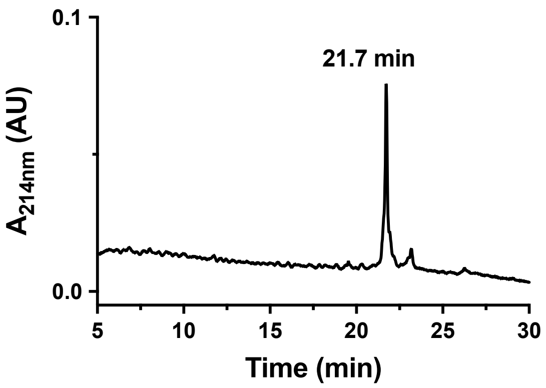

B) ESI MS (+)

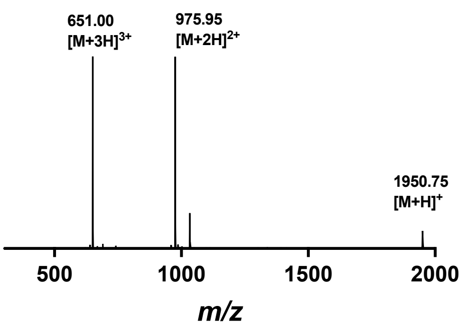

CP3

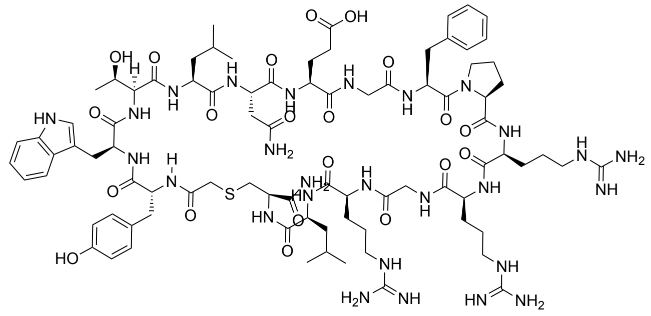

The linear peptide (50 μmol) was synthesized according to general procedure A before being cleaved from resin by general procedure C. The crude linear peptide was then purified by preparative RP-HPLC (0 vol.% CH_3CN + 0.1 vol.% TFA for 5 min, then 0-60 vol.% CH_3CN + 0.1 vol.% TFA over 80 min, 38 mL/min, XBridge® C18, 300 Å, 30 x 150 mm). The peptide was then cyclised by general procedure E before purification of the cyclic peptide by preparative HPLC (0 vol.% CH_3CN + 0.1 vol.% TFA for 10 min, then 0-60 vol.% CH_3CN + 0.1 vol.% TFA over 40 min, 38 mL/min, XBridge® C18, 300 Å, 30 x 150 mm) to afford ADO-L-03 as a white solid (11.33 mg, 12%). **A)** Analytical HPLC: R_t $_{214nm}$: 17.9 min. (1 to 60 vol.% CH_3CN+ 0.1 vol.% TFA over 30 min). **B)** MS (+ESI): m/z = 1907.4 $[M+H]^+$, 954.3 $[M+2H]^{2+}$, 636.6 $[M+3H]^{3+}$.

A) Analytical HPLC Trace (λ= 214 nm)

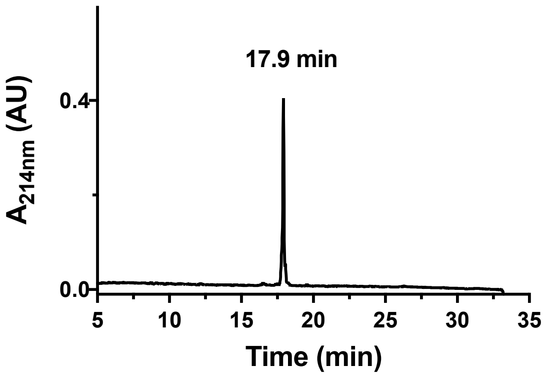

B) ESI MS (+)

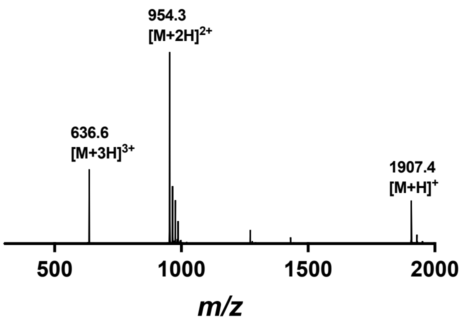

CP4

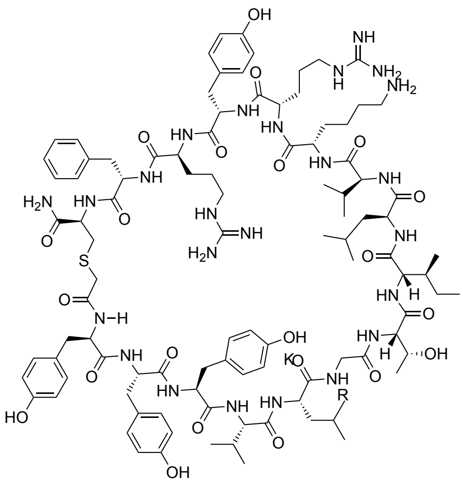

The linear peptide (50 µmol) was synthesized according to general procedure A before being cleaved from resin by general procedure C. The crude linear peptide was then purified by preparative RP-HPLC (0 vol.% CH_3CN + 0.1 vol.% TFA for 5 min, then 0-60 vol.% CH_3CN + 0.1 vol.% TFA over 80 min, 38 mL/min, XBridge® C18, 300 Å, 30 x 150 mm). The peptide was then cyclised by general procedure E before purification of the cyclic peptide by preparative HPLC (0 vol.% CH_3CN + 0.1 vol.% TFA for 10 min, then 0-60 vol.% CH_3CN + 0.1 vol.% TFA over 40 min, 38 mL/min, XBridge® C18, 300 Å, 30 x 150 mm) to afford ADO-L-04 as a white solid (0.82 mg, 0.8%). **A)** Analytical HPLC: $R_{t\ 214nm}$: 20.1 min. (1 to 60 vol.% CH_3CN+ 0.1 vol.% TFA over 30 min). **B)** MS (+ESI): m/z = 1049.00 $[M+2H]^{2+}$, 699.7 $[M+3H]^{3+}$.

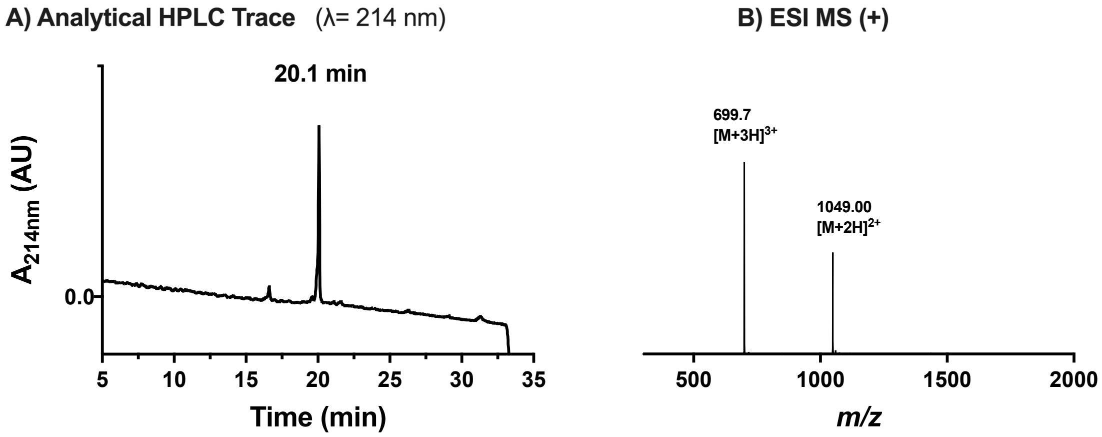

CP5

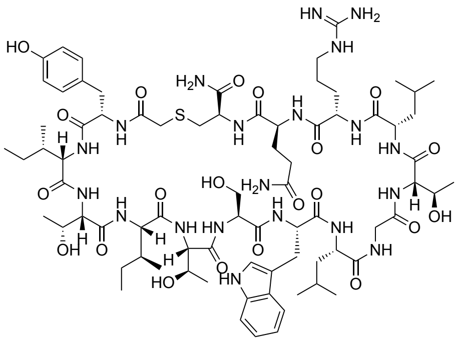

The linear peptide (50 µmol) was synthesized according to general procedure A before being cleaved from resin by general procedure C. The crude linear peptide was then purified by preparative RP-HPLC (0 vol.% CH_3CN + 0.1 vol.% TFA for 5 min, then 0-60 vol.% CH_3CN + 0.1 vol.% TFA over 80 min, 38 mL/min, XBridge® C18, 300 Å, 30 x 150 mm). The peptide was then cyclised by general procedure E before purification of the cyclic peptide by preparative HPLC (0 vol.% CH_3CN + 0.1 vol.% TFA for 10 min, then 0-60 vol.% CH_3CN + 0.1 vol.% TFA over 40 min, 38 mL/min, XBridge® C18, 300 Å, 30 x 150 mm) to afford ADO-L-05 as a white solid (3.5 mg, 4%). **A)** Analytical HPLC: $R_{t\,214nm}$: 19.5 min. (1 to 60 vol.% CH_3CN+ 0.1 vol.% TFA over 30 min). **B)** MS (+ESI): m/z = 1694.40 $[M+H]^+$, 847.75 $[M+2H]^{2+}$

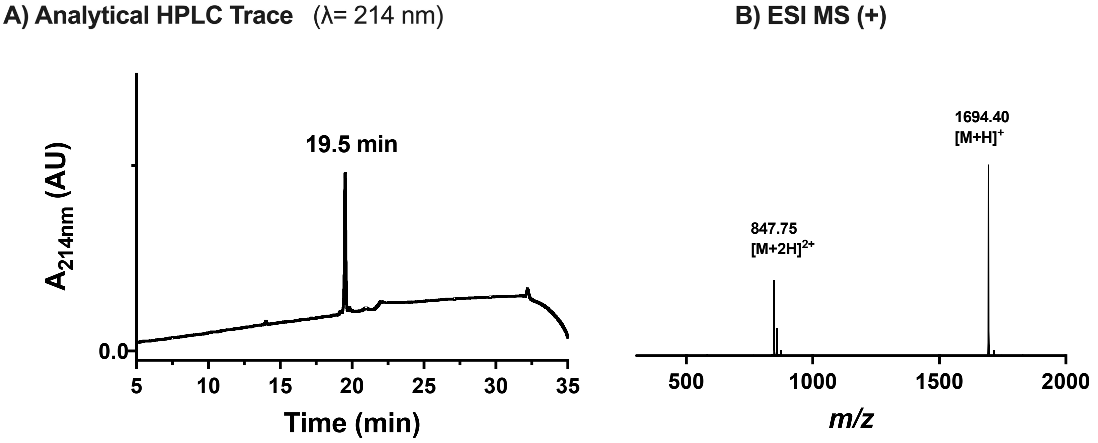

CP6

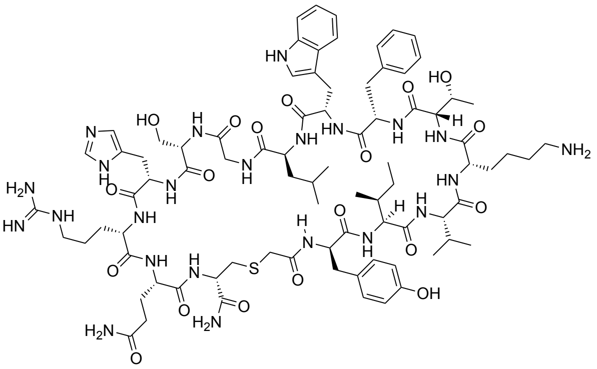

The linear peptide (50 µmol) was synthesized according to general procedure A before being cleaved from resin by general procedure C. The crude linear peptide was then purified by preparative RP-HPLC (0 vol.% CH_3CN + 0.1 vol.% TFA for 5 min, then 0-60 vol.% CH_3CN + 0.1 vol.% TFA over 80 min, 38 mL/min, XBridge® C18, 300 Å, 30 x 150 mm). The peptide was then cyclised by general procedure E before purification of the cyclic peptide by preparative HPLC (0 vol.% CH_3CN + 0.1 vol.% TFA for 10 min, then 0-60 vol.% CH_3CN + 0.1 vol.% TFA over 40 min, 38 mL/min, XBridge® C18, 300 Å, 30 x 150 mm) to afford ADO-L-06 as a white solid (7.5 mg, 8.4%). **A)** Analytical HPLC: $R_{t\ 214nm}$: 16.2 min. (1 to 60 vol.% CH_3CN+ 0.1 vol.% TFA over 30 min). **B)** MS (+ESI): m/z = 1777.40 $[M+H]^+$, 889.35 $[M+2H]^{2+}$, 593.20 $[M+3H]^3$.

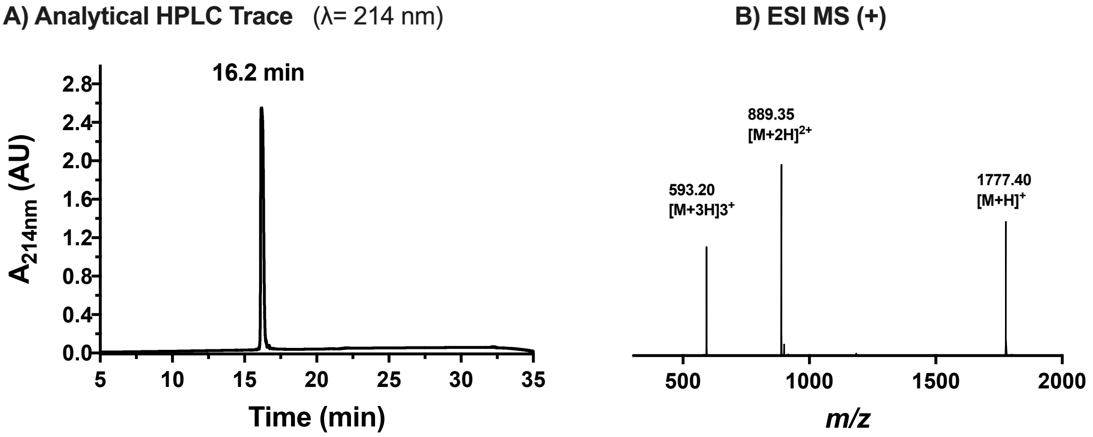

CP7

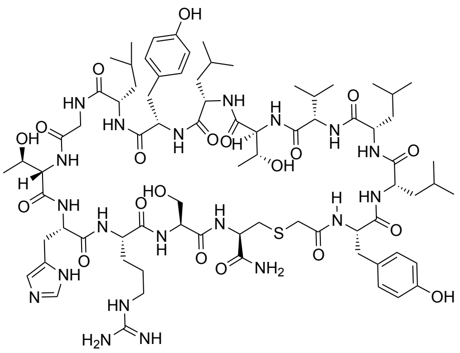

The linear peptide (50 µmol) was synthesized according to general procedure A before being cleaved from resin by general procedure C. The crude linear peptide was then purified by preparative RP-HPLC (0 vol.% CH_3CN + 0.1 vol.% TFA for 5 min, then 0-60 vol.% CH_3CN + 0.1 vol.% TFA over 80 min, 38 mL/min, XBridge® C18, 300 Å, 30 x 150 mm). The peptide was then cyclised by general procedure E before purification of the cyclic peptide by preparative HPLC (0 vol.% CH_3CN + 0.1 vol.% TFA for 10 min, then 0-60 vol.% CH_3CN + 0.1 vol.% TFA over 40 min, 38 mL/min, XBridge® C18, 300 Å, 30 x 150 mm) to afford ADO-L-01 as a white solid (4.48 mg, 5.3%). **A)** Analytical HPLC: $R_{t\ 214nm}$: 22.1 min. (1 to <u>60</u> vol.% CH_3CN+ 0.1 vol.% TFA over 30 min). **B)** MS (+ESI): m/z = 1678.40 $[M+H]^+$, 839.80 $[M+2H]^{2+}$.

A) Analytical HPLC Trace (λ= 214 nm)

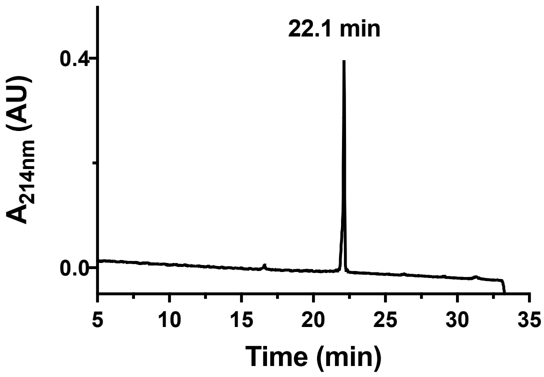

B) ESI MS (+)

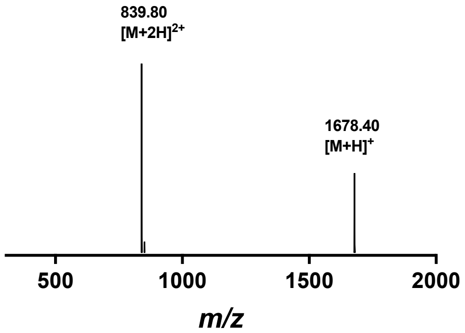

CP8

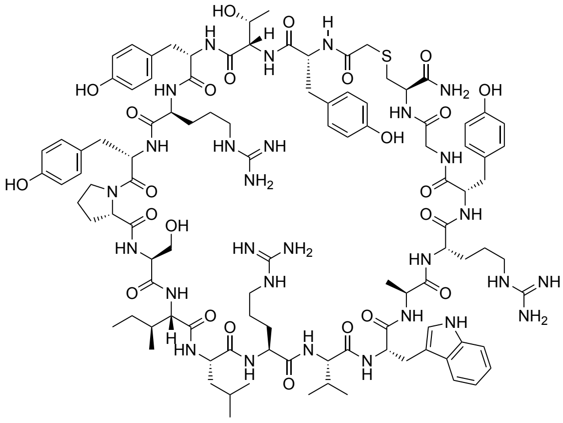

The linear peptide (50 µmol) was synthesized according to general procedure A before being cleaved from resin by general procedure C. The crude linear peptide was then purified by preparative RP-HPLC (0 vol.% CH_3CN + 0.1 vol.% TFA for 5 min, then 0-60 vol.% CH_3CN + 0.1 vol.% TFA over 80 min, 38 mL/min, XBridge® C18, 300 Å, 30 x 150 mm). The peptide was then cyclised by general procedure E before purification of the cyclic peptide by preparative HPLC (0 vol.% CH_3CN + 0.1 vol.% TFA for 10 min, then 0-60 vol.% CH_3CN + 0.1 vol.% TFA over 40 min, 38 mL/min, XBridge® C18, 300 Å, 30 x 150 mm) to afford ADO-L-08 as a white solid (20 mg, 18%). **A)** Analytical HPLC: $R_{t\,214nm}$: 20.5 min. (1 to 60 vol.% CH_3CN+ 0.1 vol.% TFA over 30 min). **B)** MS (+ESI): m/z = 1104.00 $[M+2H]^{2+}$, 736.4 $[M+3H]^{3+}$.

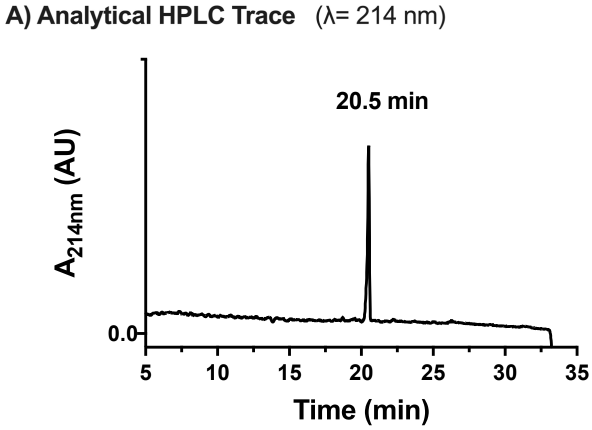

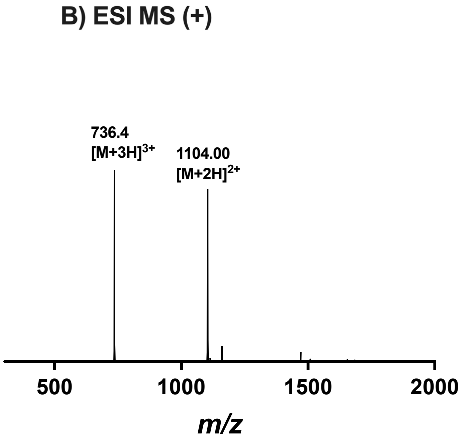

CP6-F6S

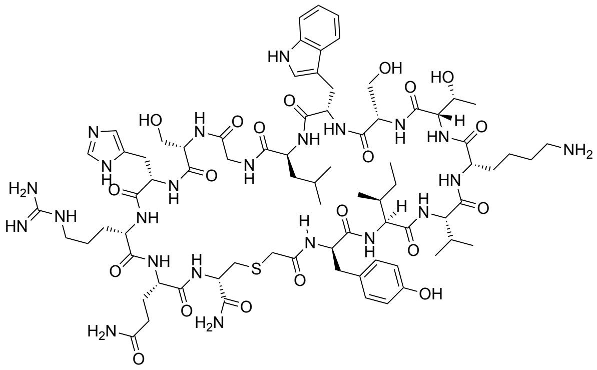

The linear peptide (50 µmol) was synthesized according to general procedure A before being cleaved from resin by general procedure C. The crude linear peptide was then purified by preparative RP-HPLC (0 vol.% CH_3CN + 0.1 vol.% TFA for 5 min, then 0-60 vol.% CH_3CN + 0.1 vol.% TFA over 80 min, 38 mL/min, XBridge® C18, 300 Å, 30 x 150 mm). The peptide was then cyclised by general procedure E before purification of the cyclic peptide by preparative HPLC (0 vol.% CH_3CN + 0.1 vol.% TFA for 10 min, then 0-60 vol.% CH_3CN + 0.1 vol.% TFA over 40 min, 38 mL/min, XBridge® C18, 300 Å, 30 x 150 mm) to afford ADO-L-06 as a white solid (3.0 mg, 3%). **A)** Analytical HPLC: $R_{t\,214nm}$: 15.6 min. (1 to 60 vol.% CH_3CN+ 0.1 vol.% TFA over 30 min). **B)** MS (+ESI): m/z = 1718.00 $[M+H]^+$, 859.30 $[M+2H]^{2+}$, 573.15 $[M+3H]^3$.

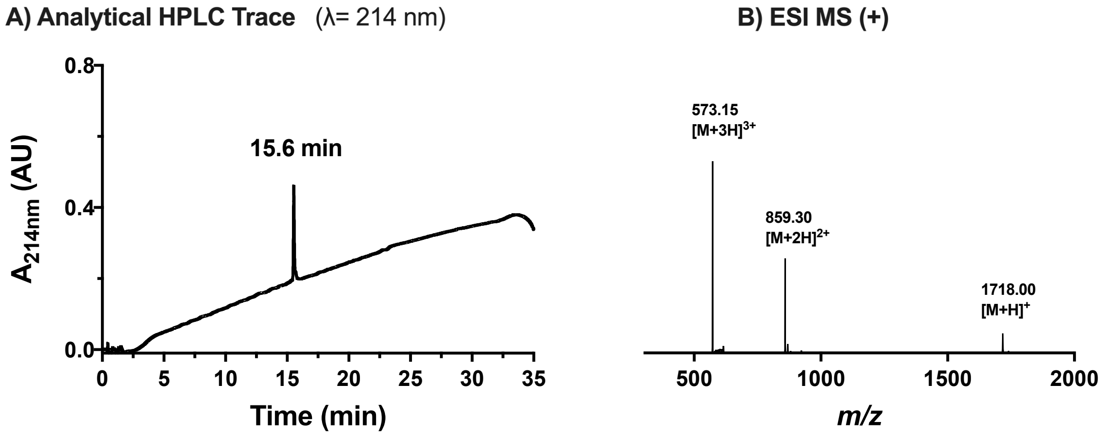

CP6-K4A

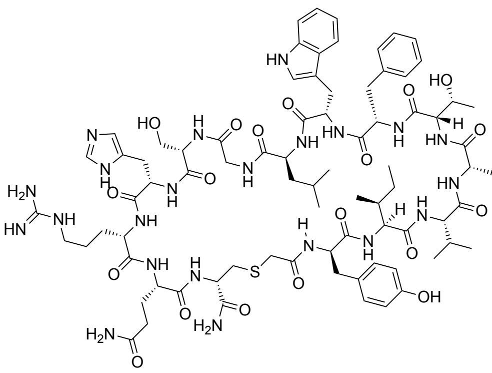

The linear peptide (50 µmol) was synthesized according to general procedure A before being cleaved from resin by general procedure C. The crude linear peptide was then purified by preparative RP-HPLC (0 vol.% CH_3CN + 0.1 vol.% TFA for 5 min, then 0-60 vol.% CH_3CN + 0.1 vol.% TFA over 80 min, 38 mL/min, XBridge® C18, 300 Å, 30 x 150 mm). The peptide was then cyclised by general procedure E before purification of the cyclic peptide by preparative HPLC (0 vol.% CH_3CN + 0.1 vol.% TFA for 10 min, then 0-60 vol.% CH_3CN + 0.1 vol.% TFA over 40 min, 38 mL/min, XBridge® C18, 300 Å, 30 x 150 mm) to afford ADO-L-06 as a white solid (2.5 mg, 3%). **A)** Analytical HPLC: $R_{t\,214nm}$: 15.6 min. (1 to 60 vol.% CH_3CN+ 0.1 vol.% TFA over 30 min). **B)** MS (+ESI): m/z = 1718.00 $[M+H]^+$, 859.30 $[M+2H]^{2+}$, 573.15 $[M+3H]^3$.

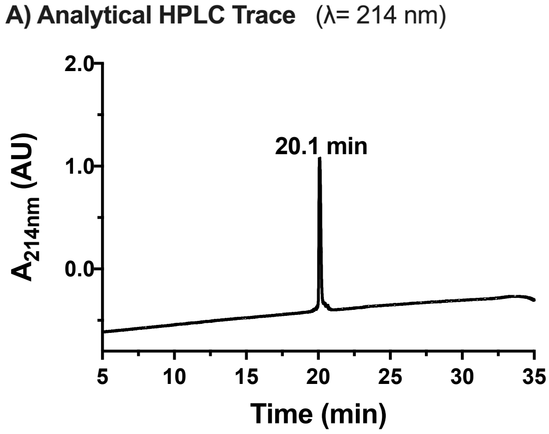

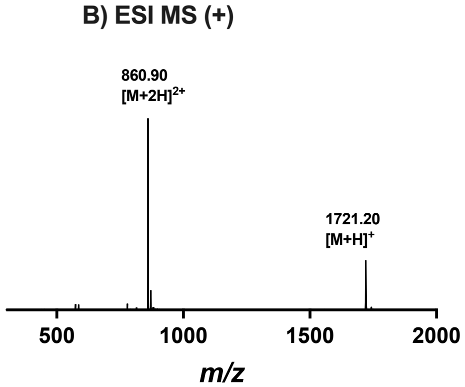

CP6-L8O-Cys

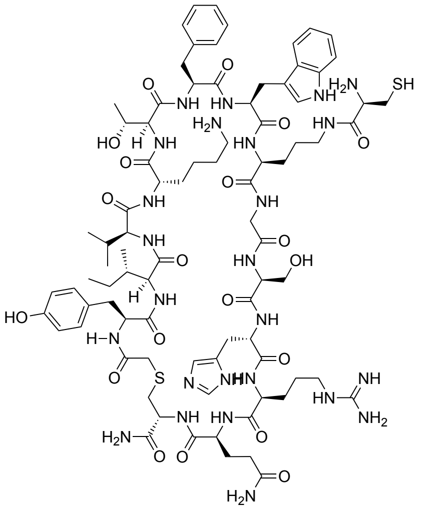

The peptide (25 µmol) was synthesised by general procedure A. The resin was then washed with CH_2Cl_2 (10 x 5 mL) before treatment with $Pd(PPh_3)_4$ (24 mg, 10 µmol, 0.2 equiv.) and $PhSiH_3$ (370 µL, 3 mmol, 30 equiv.) for 1 h, twice. The resin was then washed with CH_2Cl_2 (10 x 5 mL) and DMF (5 x 5 mL) before being treated with Fmoc-Cys(Acm)-OH (167 mg, 400 µmol), *N,N*-diisopropylcarbodiimide (63 µL, 400 µmol) and Oxyma Pure (57 mg, 400 µmol) for 16 h at rt. The resin was then washed with DMF (5 x 5 mL) CH_2Cl_2 (5 x 5 mL) and DMF (5 x 5 mL) before being treated with 2 vol.% DBU in DMF (5 mL) for 5 mins. The resin was then washed with DMF (5 x 5 mL) and CH_2Cl_2 (10 x 5 mL) before cleavage of the peptide from resin by general procedure C. The linear peptide was then purified by preparative reverse-phase HPLC and the fractions containing the desired peptide combined and cyclised by general procedure E before being lyophilized. The lyophilized peptide was then redissolved in 1:1 v/v MeCN:H_2O containing 0.1 vol.% TFA (15 mL) and treated with AgOAc (890 mg, 5.3 mmol) and stirred protected from light at room temperature for 48 h. The reaction mixture was then diluted with 1:1 v/v MeCN:H_2O, 0.1 vol.% TFA to 90 mL before addition of dithiothreitol (817 mg, 5.3 mmol). The resulting precipitate was removed by centrifugation and the supernatant collected and lyophilized. The crude peptide was then purified by reverse-phase HPLC to yield the pure peptide as a white fluffy solid (1.8 mg, 0.8%). **A)** Analytical HPLC: $R_{t\ 214nm}$: 21.5 min. (1 to 50 vol.% CH_3CN+ 0.1 vol.% TFA over 30 min). **B)** MS (+ESI) m/z = 1882.6 $[M+H]^{+}$, 941.6 $[M+2H]^{2+}$, 628.0 $[M+3H]^{3+}$, 471.2 $[M+4H]^{4+}$

A) Analytical HPLC Trace (λ= 214 nm)

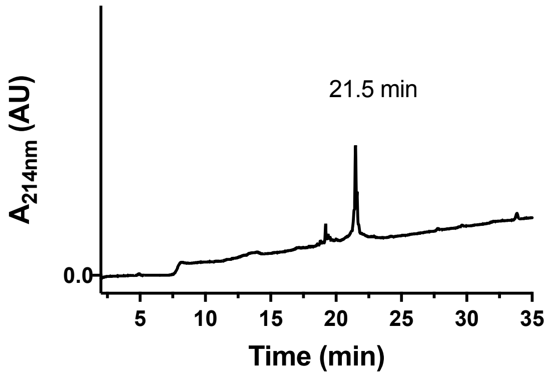

B) ESI MS (+)

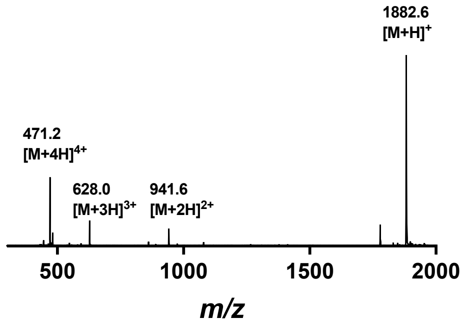

CP6-L8O-Ser

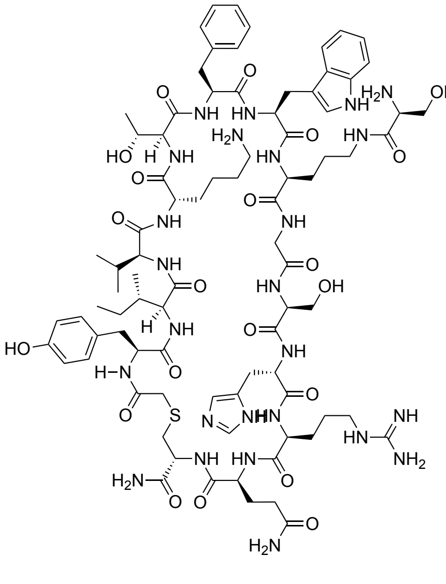

The peptide (25 µmol) was synthesised by general procedures **A**. The resin was then washed with CH_2Cl_2 (10 x 5 mL) before treatment with $Pd(PPh_3)_4$ (24 mg, 10 µmol, 0.2 equiv.) and $PhSiH_3$ (370 µL, 3 mmol, 30 equiv.) for 1 h, twice. The resin was then washed with CH_2Cl_2 (10 x 5 mL) and DMF (5 x 5 mL) before being treated with Fmoc-Ser(tBu)-OH (167 mg, 400 µmol), *N,N*-diisopropylcarbodiimide (63 µL, 400 µmol) and Oxyma Pure (57 mg, 400 µmol) for 16 h at rt. The resin was then washed with DMF (5 x 5 mL) CH_2Cl_2 (5 x 5 mL) and DMF (5 x 5 mL) before being treated with 2 vol.% DBU in DMF (5 mL) for 5 mins. The resin was then washed with DMF (5 x 5 mL) and CH_2Cl_2 (10 x 5 mL) before cleavage of the peptide from resin by general procedure C and cyclised by general procedure D. The crude peptide was then purified by reverse-phase HPLC to yield the pure peptide as a white fluffy solid (1.0 mg, 1.8%). **A)** Analytical HPLC: $R_{t\ 214nm}$: 19 min. (1 to 50 vol.% CH_3CN+ 0.1 vol.% TFA over 30 min). **B)** MS (+ESI) m/z = 1866.4 $[M+H]^+$, 467.2 $[M+4H]^{4+}$

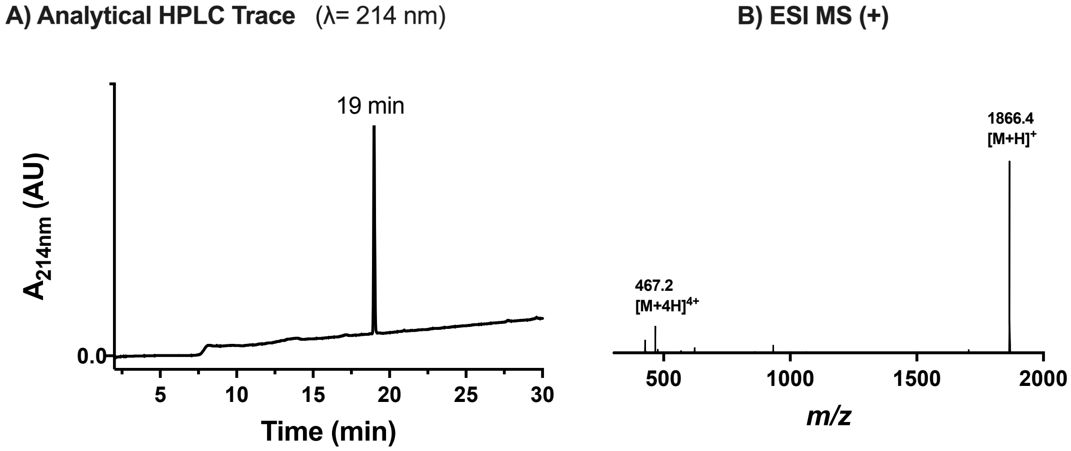

CP6-L8K-Cys

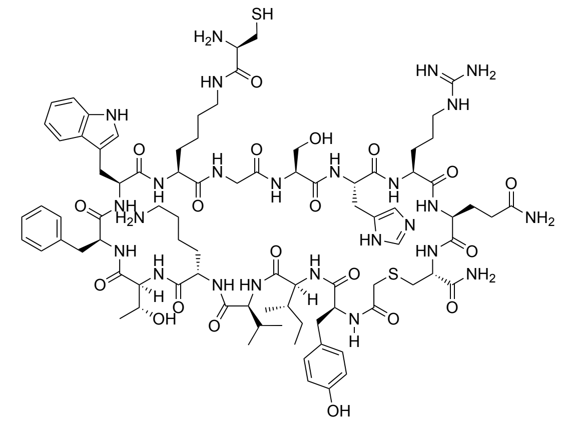

The peptide (25 μmol) was synthesised by general procedures **A**. The resin was then washed with CH_2Cl_2 (10 x 5 mL) before treatment with $Pd(PPh_3)_4$ (24 mg, 10 μmol, 0.2 equiv.) and $PhSiH_3$ (370 μL, 3 mmol, 30 equiv.) for 1 h, twice. The resin was then washed with CH_2Cl_2 (10 x 5 mL) and DMF (5 x 5 mL) before being treated with Fmoc-Cys(Acm)-OH (167 mg, 400 μmol), *N,N*-diisopropylcarbodiimide (63 μL, 400 μmol) and Oxyma Pure (57 mg, 400 μmol) for 16 h at rt. The resin was then washed with DMF (5 x 5 mL) CH_2Cl_2 (5 x 5 mL) and DMF (5 x 5 mL) before being treated with 2 vol.% DBU in DMF (5 mL) for 5 mins. The resin was then washed with DMF (5 x 5 mL) and CH_2Cl_2 (10 x 5 mL) before cleavage of the peptide from resin by general procedure **B**. The linear peptide was then purified by preparative reverse-phase HPLC and the fractions containing the desired peptide combined and lyophilized. The lyophilized peptide was then redissolved in 1:1 v/v MeCN:H_2O containing 0.1 vol.% TFA (15 mL) and treated with AgOAc (890 mg, 5.3 mmol) and stirred protected from light at room temperature for 48 h. The reaction mixture was then diluted with 1:1 v/v MeCN:H_2O, 0.1 vol.% TFA to 90 mL before addition of dithiothreitol (817 mg, 5.3 mmol). The resulting precipitate was removed by centrifugation and the supernatant collected and lyophilized. The crude peptide was then purified by reverse-phase HPLC to yield the pure peptide as a white fluffy solid (0.7 mg, 1.2%). **A)** Analytical HPLC: $R_{t\,214nm}$: 21.4 min. (1 to 50 vol.% CH_3CN+ 0.1 vol.% TFA over 30 min). **B)** MS (+ESI) m/z = 1896.5 $[M+H]^+$, 474.8 $[M+4H]^{4+}$

A) Analytical HPLC Trace (λ= 214 nm)

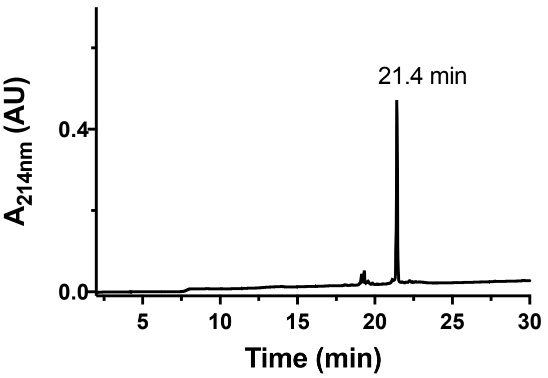

B) ESI MS (+)

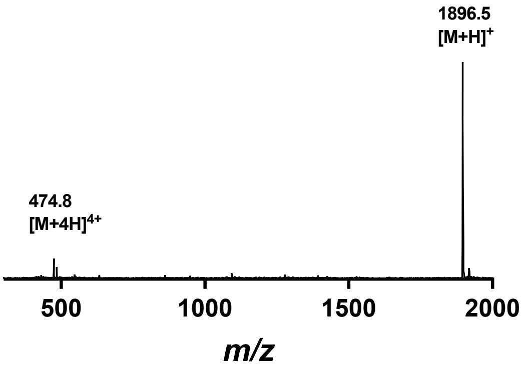

CP6-L8K-Ser

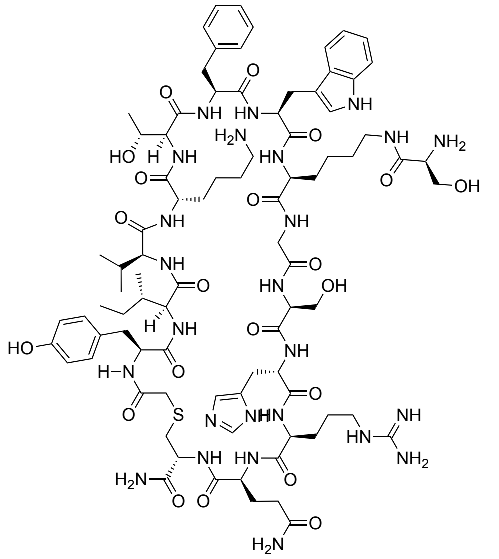

The peptide (25 µmol) was synthesised by general procedures **A**. The resin was then washed with CH_2Cl_2 (10 x 5 mL) before treatment with $Pd(PPh_3)_4$ (24 mg, 10 µmol, 0.2 equiv.) and $PhSiH_3$ (370 µL, 3 mmol, 30 equiv.) for 1 h, twice. The resin was then washed with CH_2Cl_2 (10 x 5 mL) and DMF (5 x 5 mL) before being treated with Fmoc-Ser(tBu)-OH (167 mg, 400 µmol), *N,N*-diisopropylcarbodiimide (63 µL, 400 µmol) and Oxyma Pure (57 mg, 400 µmol) for 16 h at rt. The resin was then washed with DMF (5 x 5 mL) CH_2Cl_2 (5 x 5 mL) and DMF (5 x 5 mL) before being treated with 2 vol.% DBU in DMF (5 mL) for 5 mins. The resin was then washed with DMF (5 x 5 mL) and CH_2Cl_2 (10 x 5 mL) before cleavage of the peptide from resin by general procedure **C** and cyclised by general procedure **D**. The crude peptide was then purified by reverse-phase HPLC to yield the pure peptide as a white fluffy solid (0.8 mg, 1.4%). **A)** Analytical HPLC: $R_{t\ 214nm}$: 19 min. (1 to 50 vol.% CH_3CN+ 0.1 vol.% TFA over 30 min). **B)** MS (+ESI) **B)** MS (+ESI): m/z = 1880.4 $[M+H]^{+}$, 470.6 $[M+4H]^{4+}$

A) Analytical HPLC Trace (λ= 214 nm)

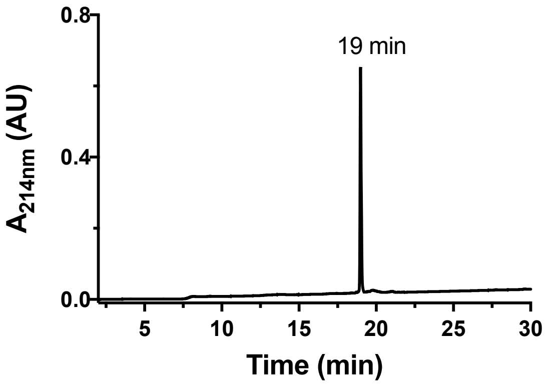

B) ESI MS (+)

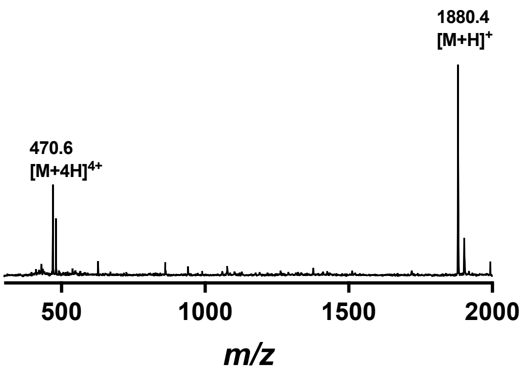

CP6-L8d-Gly-Ser

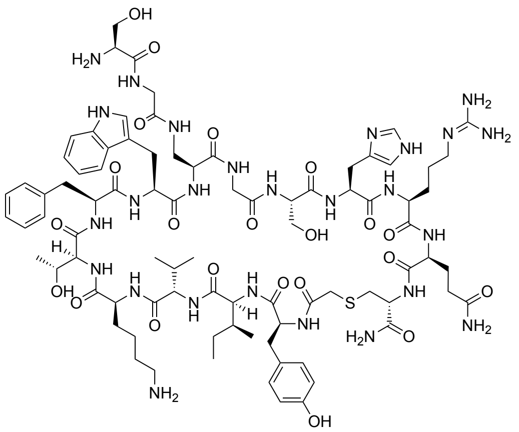

Synthesis of CP6-L8d-Gly-Ser was achieved *via* the methods outlined above. In brief, the peptide was extended as normal before DDE-L-Dap(Fmoc)-OH (CAS# 1263046-98-5) was coupled as the 7th residue and an isopeptide was extended with couplings of Fmoc-Gly-OH and then Boc-Ser(tBu)-OH. The resin bound peptide was removed of its DDE group and the remainder of the peptide extended by automated SPPS as outlined above. Following acidolytic cleavage and macrocyclization, the peptide was isolated as a white lyophilized solid (5.0 mg, 4.3% yield). **A)** Analytical HPLC: R_t 13.8 min (0 to 50% B over 30 min, λ = 214 nm) **B)** MS (+ ESI): $[M+H]^{+}$: 1894.0, $[M+2H]^{2+}$: 947.6, $[M+3H]^{3+}$: 632.1, $[M+4H]^{4+}$: 474.3.

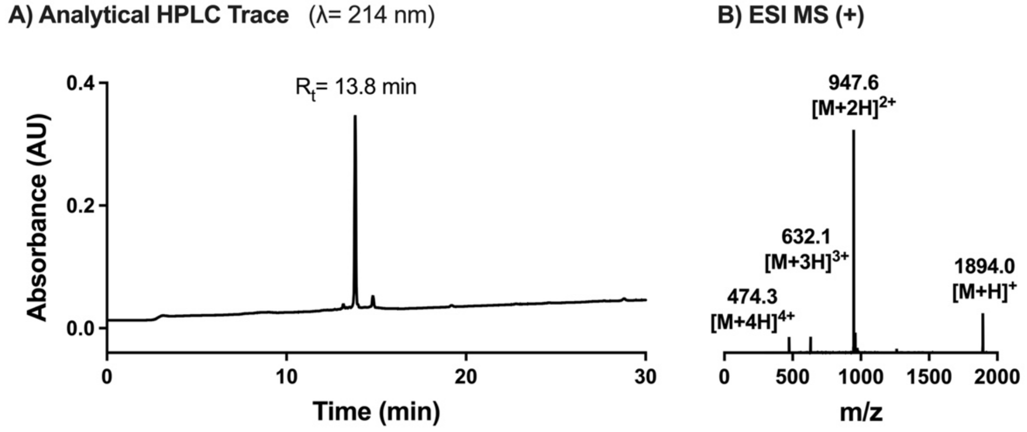

CP6-L8d-Phe-Ser

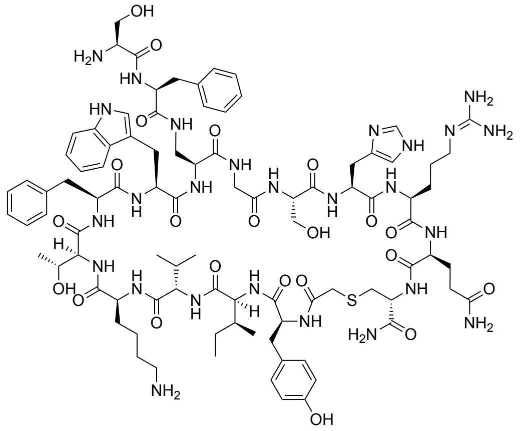

Synthesis of macrocycle CP6-L8d-Phe-Ser was achieved *via* the methods outlined above. In brief, the peptide was extended as normal before DDE-L-Dap(Fmoc)-OH (CAS# 1263046-98-5) was coupled as the 7th residue and an isopeptide was extended with couplings of Fmoc-Phe-OH and then Boc-Ser(tBu)-OH. The resin bound peptide was removed of its DDE group and the remainder of the peptide extended by automated SPPS as outlined above. Following acidolytic cleavage and macrocyclization, the peptide was isolated as a white lyophilized solid (8.2 mg, 6.7% yield). **A)** Analytical HPLC: R_t 14.8 min (0 to 50% B over 30 min, λ = 214 nm) **B)** MS (+ESI) $[M+H]^+$: 1984.2, $[M+2H]^{2+}$: 992.7, $[M+3H]^{3+}$: 662.2, $[M+4H]^{4+}$: 496.9.

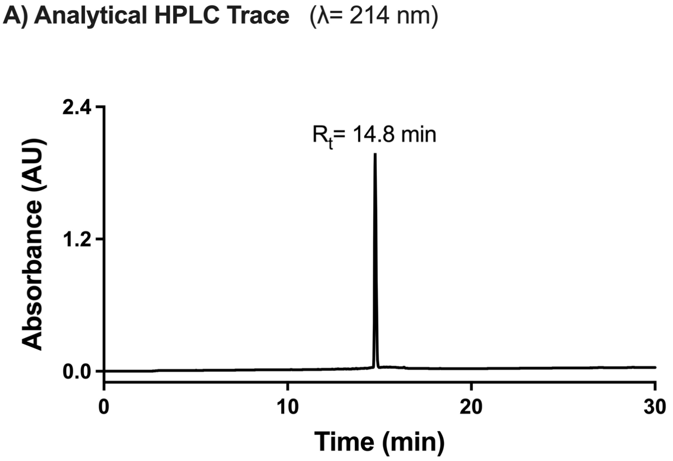

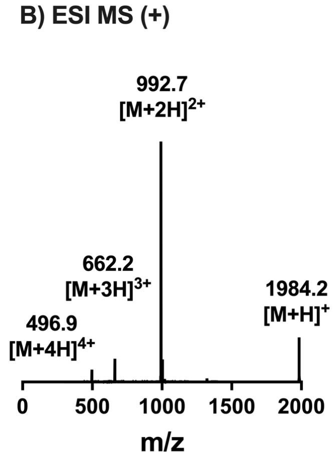

CP6-L8d-Lys-Ser

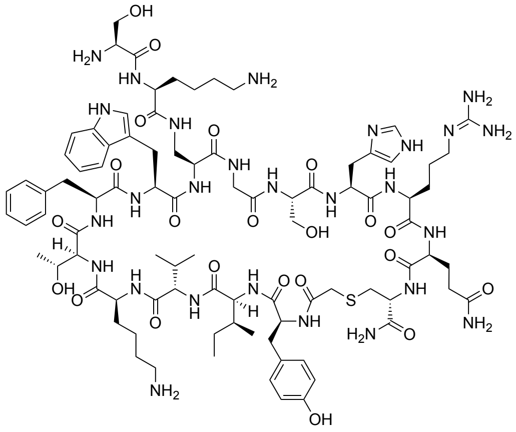

Synthesis of macrocycle CP6-L8d-Lys-Ser was achieved *via* the methods outlined above. In brief, the peptide was extended as normal before DDE-L-Dap(Fmoc)-OH (CAS# 1263046-98-5) was coupled as the 7th residue and an isopeptide was extended with couplings of Fmoc-Lys(Boc)-OH and then Boc-Ser(tBu)-OH. The resin bound peptide was removed of its DDE group and the remainder of the peptide extended by automated SPPS as outlined above. Following acidolytic cleavage and macrocyclization, the peptide was isolated as a white lyophilized solid (9.0 mg, 7.1% yield). **A)** Analytical HPLC: R_t 14.0 min (0 to 50% B over 30 min, λ = 214 nm) **B)** MS (+ESI): $[M+H]^+$: 1965.2, $[M+2H]^{2+}$: 983.2, $[M+3H]^{3+}$: 655.9, $[M+4H]^{4+}$: 492.1.

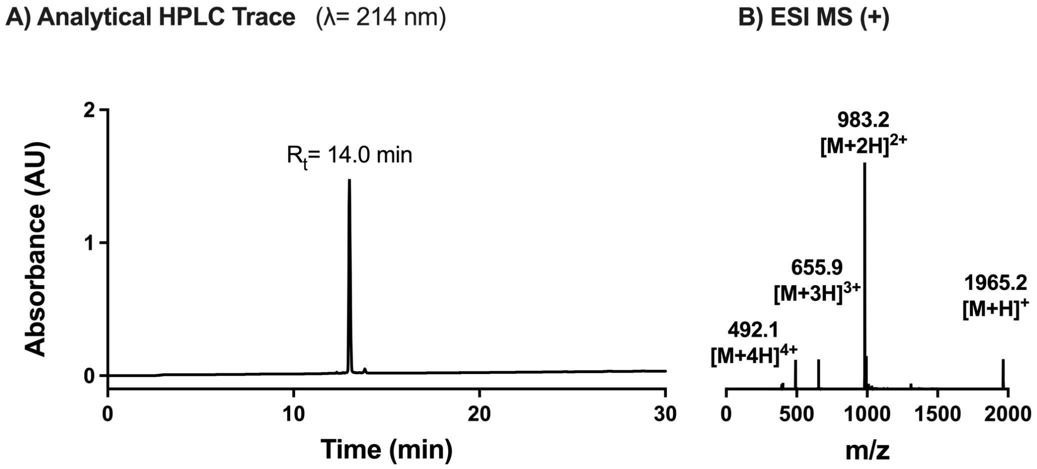

Supplement: Supplementary file 1 — Supporting information [file 41467_2025_59960_MOESM1_ESM.pdf]
