# Supplementary material for: An mRNA-display derived cyclic peptide scaffold reveals the substrate binding interactions of an N-terminal cysteine oxidase
Source: Nat Commun. 2025 May 22;16:4761. doi: 10.1038/s41467-025-59960-3 (PMC12098869; doi:10.1038/s41467-025-59960-3)
Supplement: Supplementary file 2 — Description of Additional Supplementary Files [file 41467_2025_59960_MOESM2_ESM.pdf]

Description of Additional Supplementary Files

Supplementary dataset 1

The top 100 peptide sequences, and their associated frequencies, from selection round 3 of RaPID.

Supplementary dataset 2

The top 100 peptide sequences, and their associated frequencies, from selection round 6 of RaPID.
