# Supplementary material for: An mRNA-display derived cyclic peptide scaffold reveals the substrate binding interactions of an N-terminal cysteine oxidase
Source: Nat Commun. 2025 May 22;16:4761. doi: 10.1038/s41467-025-59960-3 (PMC12098869; doi:10.1038/s41467-025-59960-3)
Supplement: Supplementary file 5 — Reporting Summary [file 41467_2025_59960_MOESM5_ESM.docx]

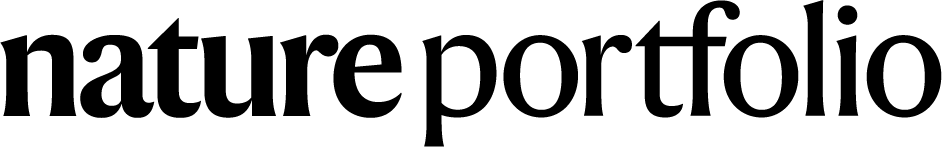
Corresponding author(s): Mark White

Last updated by author(s): 02/04/2025

Reporting Summary

Nature Portfolio wishes to improve the reproducibility of the work that we publish. This form provides structure for consistency and transparency in reporting. For further information on Nature Portfolio policies, see our Editorial Policies and the Editorial Policy Checklist.

Please do not complete any field with "not applicable" or n/a. Refer to the help text for what text to use if an item is not relevant to your study. For final submission: please carefully check your responses for accuracy; you will not be able to make changes later.

## Statistics

For all statistical analyses, confirm that the following items are present in the figure legend, table legend, main text, or Methods section.

n/a


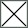

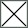

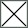

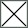

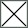

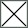

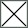


Confirmed


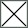
 The exact sample size (*n*) for each experimental group/condition, given as a discrete number and unit of measurement


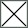
 A statement on whether measurements were taken from distinct samples or whether the same sample was measured repeatedly The statistical test(s) used AND whether they are one- or two-sided

*Only common tests should be described solely by name; describe more complex techniques in the Methods section.*


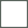
 A description of all covariates tested


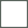
 A description of any assumptions or corrections, such as tests of normality and adjustment for multiple comparisons

A full description of the statistical parameters including central tendency (e.g. means) or other basic estimates (e.g. regression coefficient) AND variation (e.g. standard deviation) or associated estimates of uncertainty (e.g. confidence intervals)

For null hypothesis testing, the test statistic (e.g. *F*, *t*, *r*) with confidence intervals, effect sizes, degrees of freedom and *P* value noted

1

nature portfolio | reporting summary

*April 2023*

*Give P values as exact values whenever suitable.*

For Bayesian analysis, information on the choice of priors and Markov chain Monte Carlo settings

For hierarchical and complex designs, identification of the appropriate level for tests and full reporting of outcomes Estimates of effect sizes (e.g. Cohen's *d*, Pearson's *r*), indicating how they were calculated

*Our web collection on statistics for biologists contains articles on many of the points above.*

## Software and code

Policy information about availability of computer code Data collection

SPR data were collected using a Biacore T200 instrument (Cytiva). Activity data were collected using an ExionLC AD (Sciex) and SelexION (Sciex) mass spectrometer. Instrument parameters, data acquisition and data processing were controlled by Analyst software (Sciex). X-ray diffraction data were collected at the Australian Synchrotron using the Macromolecular Crystallography MX1 (bending magnet) and MX2 (microfocus) beamlines.

Data analysis

SPR data was analysed using Biacore Insight Evaluation Software. Activity data was processed using Skyline software 54. All figures and parameters were generated using GraphPad Prism 9. X-ray diffraction data were integrated using XDS and processed further using the CCP4i suite. AIMLESS was used for indexing, scaling, and merging of the data and the initial phases were calculated using PhaserMR. Manual model building was performed using COOT and refinement was performed using Phenix. Structure diagrams were generated using CCP4MG and PyMOL. Molecular dynamics simulations were conducted using Maestro, Schrödinger.

For manuscripts utilizing custom algorithms or software that are central to the research but not yet described in published literature, software must be made available to editors and reviewers. We strongly encourage code deposition in a community repository (e.g. GitHub). See the Nature Portfolio guidelines for submitting code & software for further information.

2

nature portfolio | reporting summary

*April 2023*

## Data

Policy information about availability of data

All manuscripts must include a data availability statement. This statement should provide the following information, where applicable:

- Accession codes, unique identifiers, or web links for publicly available datasets
- A description of any restrictions on data availability
- For clinical datasets or third party data, please ensure that the statement adheres to our policy

The authors confirm that all data supporting the findings presented in this study are available in the article and its supplementary sections. Source data are provided with this paper. The coordinates of cobalt-incorporated ADO in complex with CP6 were deposited to the Protein Data Bank (PDB) under accession code 9DXU [https://doi.org/10.2210/pdb9DXU/pdb]. The coordinates of cobalt-incorporated ADO in complex with CP6-L8K-Ser were deposited to the PDB under accession code 9DXV [https://doi.org/10.2210/pdb9DXV/pdb]. The coordinates of cobalt-incorporated ADO in complex with CP6-L8d-Gly-Ser were deposited to the PDB under accession code 9DXB [https://doi.org/10.2210/pdb9DXB/pdb]. The coordinates of cobalt-incorporated ADO in the absence of a CP were obtained from the PDB using accession code 8UAN [https://doi.org/10.2210/pdb8UAN/pdb]. The coordinates of iron-incorporated CDO in complex with Cys were obtained from the PDB using accession code 4IEV [https://doi.org/10.2210/pdb4IEV/pdb].

## Research involving human participants, their data, or biological material

Policy information about studies with human participants or human data. See also policy information about sex, gender (identity/presentation), and sexual orientation and race, ethnicity and racism.

Reporting on sex and gender

N/A

Reporting on race, ethnicity, or other socially relevant groupings

N/A

Population characteristics

N/A

Recruitment

N/A

Ethics oversight

N/A

Note that full information on the approval of the study protocol must also be provided in the manuscript.

# Field-specific reporting

Please select the one below that is the best fit for your research. If you are not sure, read the appropriate sections before making your selection.


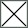
 Life sciences
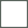
 Behavioural & social sciences
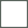
 Ecological, evolutionary & environmental sciences

For a reference copy of the document with all sections, see nature.com/documents/nr-reporting-summary-flat.pdf

# Life sciences study design

Sample size is indicated in the figure, figure legend and/or method. No statistical method was used to predetermined sample size. The data presented is biochemical and biophysical in nature.

All studies must disclose on these points even when the disclosure is negative. Sample size

Data exclusions

No technically sound data were excluded.

Replication

Experiments were replicated for the indicated number of times. This is at least 3 times for binding, activity and MD simulation experiments. All attempts to replicate the data were successful.

Randomization

R-free values were calculated using random test sets. Sample randomization was not relevant to other aspects of this study.

Blinding

Blinding was not relevant to this study. Samples were prepared and analysed by the same individual(s).

# Reporting for specific materials, systems and methods

We require information from authors about some types of materials, experimental systems and methods used in many studies. Here, indicate whether each material, system or method listed is relevant to your study. If you are not sure if a list item applies to your research, read the appropriate section before selecting a response.

3

nature portfolio | reporting summary

*April 2023*

Materials & experimental systems Methods


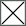

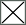

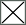

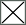

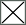

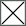

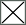


n/a Involved in the study Antibodies Eukaryotic cell lines

Palaeontology and archaeology Animals and other organisms Clinical data

Dual use research of concern

Plants


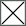

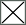

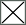


n/a Involved in the study

ChIP-seq

Flow cytometry

MRI-based neuroimaging

## Plants

Seed stocks

*Report on the source of all seed stocks or other plant material used. If applicable, state the seed stock centre and catalogue number. If plant specimens were collected from the field, describe the collection location, date and sampling procedures.*

*Describe the methods by which all novel plant genotypes were produced. This includes those generated by transgenic approaches, gene editing, chemical/radiation-based mutagenesis and hybridization. For transgenic lines, describe the transformation method, the number of independent lines analyzed and the generation upon which experiments were performed. For gene-edited lines, describe the editor used, the endogenous sequence targeted for editing, the targeting guide RNA sequence (if applicable) and how the editor was applied.*

*Describe any authentication procedures for each seed stock used or novel genotype generated. Describe any experiments used to*

*assess the effect of a mutation and, where applicable, how potential secondary effects (e.g. second site T-DNA insertions, mosiacism, off-target gene editing) were examined.*

Novel plant genotypes

Authentication


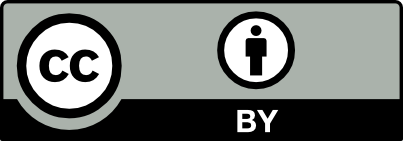
This checklist template is licensed under a Creative Commons Attribution 4.0 International License, which permits use, sharing, adaptation, distribution and reproduction in any medium or format, as long as you give appropriate credit to the original author(s) and the source, provide a link to the Creative Commons license, and indicate if changes were made. The images or other third party material in this article are included in the article's Creative Commons license, unless indicated otherwise in a credit line to the material. If material is not included in the article's Creative Commons license and your intended use is not permitted by statutory regulation or exceeds the permitted use, you will need to obtain permission directly from the copyright holder. To view a copy of this license, visit <http://creativecommons.org/licenses/by/4.0/>
